# Water, Sanitation, and Child Health: Evidence From Subnational Panel Data in 59 Countries

**DOI:** 10.1007/s13524-019-00760-y

**Published:** 2019-02-28

**Authors:** Derek Headey, Giordano Palloni

**Affiliations:** The International Food Policy Research Institute (IFPRI), 1201 Eye Street NW, Washington, DC 20005 USA

**Keywords:** Sanitation, Water, Child health, Child mortality

## Abstract

Water, sanitation, and hygiene (WASH) investments are widely seen as essential for improving health in early childhood. However, the experimental literature on WASH interventions identifies inconsistent impacts on child health outcomes, with relatively robust impacts on diarrhea and other symptoms of infection but weak and varying impacts on child nutrition. In contrast, observational research exploiting cross-sectional variation in water and sanitation access is much more sanguine, finding strong associations with diarrhea prevalence, mortality, and stunting. In practice, both literatures suffer from significant methodological limitations. Experimental WASH evaluations are often subject to poor compliance, rural bias, and short duration of exposure, while cross-sectional observational evidence may be highly vulnerable to omitted variables bias. To overcome some of the limitations of both literatures, we construct a panel of 442 subnational regions in 59 countries with multiple Demographic Health Surveys. Using this large subnational panel, we implement difference-in-difference regressions that allow us to examine whether longer-term changes in water and sanitation at the subnational level predict improvements in child morbidity, mortality, and nutrition. We find results that are partially consistent with both literatures. Improved water access is statistically insignificantly associated with most outcomes, although water piped into the home predicts reductions in child stunting. Improvements in sanitation predict large reductions in diarrhea prevalence and child mortality but are not associated with changes in stunting or wasting. We estimate that sanitation improvements can account for just under 10 % of the decline in child mortality from 1990 to 2015.

**Electronic supplementary material:**

The online version of this article (10.1007/s13524-019-00760-y) contains supplementary material, which is available to authorized users.

## Introduction

The past decade has witnessed a renewed global interest in the health impacts of improved water, sanitation, and hygiene (WASH). The Millennium Development Goal (MDG) era saw solid progress in WASH indicators, with almost 2 billion people gaining access to improved water and/or sanitation. However, some 700 million still lack access to improved water; in addition, approximately 2.5 billion people do not use an improved sanitation facility, and of these, 1 billion people still practice open defecation (WHO and UNICEF 2014).

The persistence of these problems is a significant public health concern. Human feces are an important reservoir for a range of pathogenic bacteria as well as soil-transmitted helminths (STHs) that can cause diarrhea, environmental enteric disorder (EED), trachoma, and other morbidities prevalent in young children (Mara et al. 2010). Diarrhea and EED are also thought to be important determinants of malnutrition in young children (Checkley et al. 2008; Humphrey 2009). Moreover, many of these morbidities, in combination with poor nutrition, often prove fatal if not properly treated, suggesting that poor WASH conditions could be a major underlying risk factor for child mortality (Mara et al. 2010).

Yet, despite several plausible biological pathways, the empirical evidence linking WASH conditions to child health outcomes is limited and, for some health outcomes, inconsistent. Evidence from cluster randomized control trials (RCTs) and case-control studies suggests reasonably strong and consistent impacts of WASH interventions on diarrhea incidence (Fewtrell et al. 2005; Freeman et al. 2017; Wolf et al. 2014) and STH infections (Freeman et al. 2017; Strunz et al. 2014; Ziegelbauer et al. 2012). However, RCT estimates of WASH impacts on child stunting and wasting are often statistically insignificant (Dangour et al. 2013; Freeman et al. 2017). In contrast, observational research has typically found very strong associations with child health and nutrition outcomes. A range of historical studies have linked reductions in child mortality to WASH improvements in the nineteenth and early twentieth centuries (Cutler and Miller 2005; Woods et al. 1989). For 71 contemporary developing countries, a pooled multivariate regression analysis of Demographic Health Survey (DHS) data found that household water and sanitation facilities were strongly associated with lower risk of child mortality, diarrhea, and stunting (Fink et al. 2011). Various papers by Spears and colleagues also used DHS data to link child mortality, stunting, and anemia to toilet use within the broader community, on the premise that open defecation has negative interhousehold externalities on child health (Coffey et al. 2016; Geruso and Spears 2018; Spears 2013a). This research also uncovers evidence that open defecation may have more harmful effects in densely populated regions, such as South Asia (Hathi et al. 2017; Spears 2013a).

These different literatures therefore have tended to find reasonably strong evidence of WASH impacts on diarrhea, but impacts on child nutrition and mortality outcomes remain uncertain. In practice, both the experimental and observational literatures have important methodological limitations. Several commentaries raise concerns about the quality of the RCT evidence, highlighting issues such as the low adoption of WASH interventions and the short duration of exposure to WASH treatments (Headey 2016; Huda et al. 2012; Schmidt 2014). Still, observational studies also have inherent limitations. Most have used repeated cross-sections in which WASH exposure is not clearly linked to any specific intervention and is therefore likely to be strongly correlated with a range of confounding factors, including parental knowledge and preferences, cultural norms, local economic development, historical infrastructural investments, governance quality, and environmental factors, such as population density (Coffey et al. 2017; Davis 2004; Ndikumana and Pickbourn 2017). Adequately controlling for these interhousehold and intercommunity differences with cross-sectional survey data is likely to be extremely difficult, if not impossible. As a result, it is difficult to argue that these studies convincingly inform the more policy-relevant question that experimental studies pose: do *changes* in WASH exposure lead to *changes* in child health outcomes?

In this study, we use a subnational panel data set constructed from aggregated DHS to address this important policy question. Although the DHS are not a panel of children or households, they are a panel of subnational regions, the smallest geographical unit at which the DHS are representatively sampled. Moreover, DHS data on child health, sanitation, and other determinants of child health have been collected within countries in successive DHS waves over relatively long periods. These two features allow us to construct a rich subnational panel covering 442 subnational regions in 59 countries with multiple DHS rounds, resulting in approximately 1,500 observations for mortality, diarrhea prevalence, and fever prevalence, and 1,176 observations for stunting and wasting. This data structure has several key advantages.

First and foremost, it permits controls for panel fixed effects, thereby netting out the important time-invariant confounding factors listed earlier. Thus, we estimate difference-in-difference (DID) regressions that control for any non-time-varying subnational characteristics, regardless of whether they are observable in the data.

Second, subnational data exploit the growing importance of decentralized governance in developing countries. The importance of state-level changes in WASH in India and Nepal has been well documented (Coffey et al. 2016, 2017; Spears 2013b), but there are many other subnational WASH success stories. In Ethiopia, for example, the Southern Nations, Nationalities, and Peoples’ (SNNP) regional government implemented an exceptionally rapid expansion of community-led total sanitation over 2003–2005 prior to a national scale-up in 2006 (World Bank 2007).

Third, although changes in WASH access are not random in these data, DID regressions restrict endogeneity concerns to time-varying confounding factors, which we may be better able to adequately control for by including time-varying indicators from the DHS and other sources. Moreover, panel data permit us to assess to some extent—by exploring associations between the WASH variables and other likely determinants of child health and through parallel trends exercises—how likely it is that two of the likely sources of potential bias are driving the results.

Finally, in addition to addressing issues of internal validity, the geographical spread of DHS data allows us to speak to important issues of external validity, particularly whether the health benefits of expanded WASH access vary with population density (Hathi et al. 2017) or child age (Alderman and Headey 2018).

Our results suggest that changes in subnational sanitation coverage predict sizable improvements in child morbidity and mortality. A 1 percentage point increase in sanitation coverage is associated with a decrease in under-5 child mortality of between 0.34 and 0.38 per 1,000 births and a decrease in the prevalence of diarrhea during the two weeks preceding the survey of between 0.056 and 0.12 percentage points. In contrast, we find no statistically significant association between sanitation coverage and stunting or wasting, and the association with the prevalence of fever is highly sensitive to the specification used. Combining our estimates with the observed increase in global sanitation coverage between 1990–2015 indicates that changes in sanitation coverage can potentially explain 8.2 % of the total observed decline in under-5 mortality over the same period. We find little evidence that increases in access to any improved water source—according to the official definition—are statistically significantly associated with health and nutrition improvements. However, water piped into the home predicts significant reductions in child stunting, suggesting that the official definition of “improved water” may need to be revisited.

## Materials and Methods

### Data

The DHS have now been implemented for approximately three decades and used extensively to analyze the main health outcomes in this study: child mortality, morbidity, and nutrition. As a result, many countries have multiple DHS waves, with each wave a cross-section of households rather than a panel. However, because the DHS have complex survey designs to achieve subnational representativeness, they can be aggregated into a panel of *subnational* units (states/provinces, districts, ecological zones, or simply rural and urban areas). Although these subnational units have sometimes changed within countries to become more spatially disaggregated, DHS STATcompiler (USAID and ICF-International 2017) can be used to construct a spatially consistent panel defined by earlier classifications of subnational units. This allows us to construct a panel with multiple rounds that spans relatively long periods.^1^ The panel, however, is highly unbalanced in the time dimension, both in terms of the number of surveys per country and the time interval between surveys (see Table S1 in the online appendix for survey details). Our final data set includes data from 218 DHS rounds in 59 countries drawn from four major regions/continents (Latin America, Africa, Asia, and Europe and Central Asia), with well over 1,000 observations for our main outcomes of interest.

Although the subnational STATcompiler panel we use is advantageously large and long, a potentially important disadvantage is that it does not allow for flexible age disaggregation in nutrition and health indicators, nor does it allow us to restrict the data used to calculate subnational child mortality rates.^2^ To test sensitivity to these variations, we therefore use survey weights to aggregate DHS microdata into two subnational panels to examine nutrition and morbidity associations by child age and to vary the recall period used to estimate the child mortality rates.^3^ These additional subnational panels cover most of the observations in our main STATcompiler data set, and we show in the online appendix that the change in sample does not affect our results in any material fashion. Further details of all three subnational panels are provided in Section A of the online appendix.^4^

#### Dependent Variables

The primary child health outcomes in our analysis are selected based on the outcomes typically used in the WASH literature summarized earlier: the under-5 mortality rate (per 1,000 births) based on a 10-year recall period, diarrhea prevalence in the previous two weeks, and stunting prevalence (height-for-age z score (HAZ) < –2 standard deviations). We also investigate two secondary outcomes: (1) the prevalence of child wasting (weight-for-height *Z* score < –2 standard deviations), which is often included in experimental and observational studies on WASH; and (2) fever prevalence in the previous two weeks as an additional marker of infections that might be influenced by WASH status.

#### Drinking Water and Sanitation Variables

*A priori*, it is not clear which types of WASH technologies matter most for improving specific child health outcomes. Some of the literature cited earlier has concluded that more sophisticated WASH technologies have larger health impacts, whereas others have argued that the introduction of basic WASH technologies can yield large benefits. Gunther and Fink (2010) compared and contrasted several drinking water and sanitation definitions, including private/public (shared) and technology-based definitions, and Spears (2013a) implicitly argued that the health benefits of moving from open defecation to *any* form of toilet use (fixed-point defecation) is the most critical step on the sanitation ladder because of the primary importance of negative externalities across households. Importantly, our use of subnational data captures both household-level effects and community-level externalities.

In our main specifications, we first focus on the use of “any toilet” and “any improved water,” with the latter following the definition of the WHO/UNICEF Joint Monitoring Program (JMP). However, in robustness tests, we disaggregate these measures. “Any toilet” is split into an improved category (flush/pour toilets, pit latrines with a slab or ventilated, compositing toilet) and an unimproved category consisting mostly of basic pit latrines. “Any improved water” is disaggregated based on a modification of the technological classification in Gunther and Fink (2010), which distinguished “piped to home” (dwelling, yard), “piped to other” (public tap/standpipe, neighbor), and “other improved” (a third category comprising tubewells/boreholes and protected wells/springs).

#### Control Variables

Our control variables are selected based on an assessment of commonly cited determinants of reductions in diarrhea, stunting, and mortality. These consist of subnational DHS-based indicators as well as a series of national-level controls for variables not well captured in the DHS, which we source from the World Bank (2017). DHS measures include housing characteristics, maternal education, demographic indicators, and health services. At the national level, we control for log GDP per capita, cereal yields (a food security proxy), health expenditures as a percentage of GDP, foreign aid, urbanization, population, and malaria incidence. In some specifications, we also use log population density (people per square km) measured at the subnational level as an interaction variable. This indicator draws on census data compiled by Hathi et al. (2017), supplemented by subnational population density estimates from the GRUMP (Center for International Earth Science Information Network (CIESIN) et al. 2008) database. Summary statistics for the control variables are presented in Table S3 in the online appendix.

### Methods

To estimate the impacts of changes in sanitation and water access on child health, we employ subnational region fixed-effects models that take the following form:

1 $$ {H}_{i,j,t}={\boldsymbol{\upbeta}}_W{\mathbf{W}}_{i,j,t}+{\boldsymbol{\upbeta}}_{\mathbf{X}}{\mathbf{X}}_{i,j,t}+{\boldsymbol{\upbeta}}_{\mathbf{Z}}{\mathbf{Z}}_{j,t}+{\boldsymbol{\upmu}}_{i,j}+{\boldsymbol{\upalpha}}_t+{\boldsymbol{\upgamma}}_{j,t}+{e}_{i,j,t}. $$

In this model, *H* is a health indicator for subnational unit *i* in country *j* at time *t*; **W** is a vector of corresponding water and sanitation indicators; **X** is a vector of subnational region control variables from the DHS; **Z** is a vector of country-level control variables; **μ**_*i*,*j*_ is a vector of subnational region fixed effects,; **α**_*t*_ is a full set of year fixed effects,; and **γ**_*j*,*t*_ are a set of either survey fixed effects or continent-specific linear time trends. We estimate three variations of Eq. (1). First, we estimate a naïve fixed-effects model that controls only for year fixed effects and the continent-specific time trends. Second, we estimate a model that additionally includes subnational and country-level control variables (**X** and **Z**).^5^ Finally, we estimate a more stringent model that controls for survey fixed effects instead of the continent-specific time trends. The survey fixed effects absorb any variation in a survey year that is common across all subnational regions in the country. This is advantageous in that it absorbs both unobservable national-level shocks and survey-specific anomalies such as changes in survey timings, the latter of which may be important for seasonal indicators, such as wasting, diarrhea, and fever prevalence. A potential disadvantage, however, is that these fixed effects will also absorb useful and uncontaminated variation in the indicators of interest.^6^ For all regressions, we estimate and report coefficient *p* values based on cluster-robust standard errors that allow for arbitrary within-subnational region correlation in the errors.^7^

## Results

### Descriptive Statistics

Table 1 reports various descriptive statistics for our outcomes and WASH measures. In the online appendix, Table S3 does the same for the control variables, the other potential determinants of the main outcomes that we use as dependent variables in falsification checks, and the age-disaggregated mortality measures. Table S4 does the same for the outcomes generated from the aggregated DHS microdata. The second column of Table 1 reports the number of observations by indicator. We have well in excess of 1,000 observations for all indicators, with more than 1,400 observations for mortality and morbidity estimates (a number of DHS do not record nutrition outcomes). The third column reports the intracountry variation in each indicator (the share of total variation within the panel not accounted for by country-level fixed effects) to demonstrate the importance of subnational disaggregation in key variables. Among child health outcomes, subnational variation accounts for between 36.8 % and 67.9 % of the total variation. Similarly, the WASH measures show substantial intracountry variation, suggesting considerable value to using subnational rather than country-level regressions.^8^ The other moments (mean, 25th, 50th, and 75th percentiles) illustrate cross-subnational region variation in the outcomes and WASH indicators, as expected given the highly varied levels of development in the sample. Although it is not observable in the summary statistic tables, we also find important variation in the outcomes and in WASH coverage over time within subnational regions. The largest improvements in toilet use occurred in the SNNP and Amhara regions of Ethiopia as well as various regions in Nepal, Bangladesh, and Cambodia. Interestingly, all are well-known adopters of Community Led Total Sanitation, which typically focuses on catalyzing construction of simple put latrines. Cambodia has also seen a rapid expansion in access to improved water, as have several very arid subnational regions in Chad, Burkina Faso, Niger, and Kenya that began with very poor access and saw marked improvements in access to improved tubewells. Overall, there appears to be ample variation in water and sanitation access and in the outcomes, creating the opportunity for a quasi-experimental DID analysis.

**Table 1:** Summary statistics for child health outcomes and key WASH indicators

				Percentiles		
	Number of Observations	Within-Country Variation (%)^a^	Mean	25th	50th	75th
Outcomes^b^						
Under-5 mortality rate (per 1,000 live births)	1,497	39.0	93.8	51.0	81.0	125.0
Children with diarrhea	1,547	55.6	18.8	13.0	18.0	23.9
Children stunted	1,208	40.1	35.0	25.2	35.0	44.9
Children with fever	1,606	67.9	29.8	21.9	29.1	37.3
WASH Measures^b^						
Households with any sanitation	1,548	40.0	71.9	53.3	81.2	94.7
Households with improved toilet	1,548	63.8	31.2	4.0	22.8	51.5
Households with unimproved toilet	1,548	53.8	40.7	10.6	37.3	67.5
Households with improved water source	1,612	50.9	71.7	58.3	74.8	88.3
Households with any piped water source	1,612	36.0	28.0	10.1	21.5	41.0
Households with nonpiped improved water source	1,612	45.6	43.7	28.9	42.8	56.8

^a^This indicator reports the share of total variation in the subnational panel explained by intracountry variation. It is equal to 100 minus the *R*^2^ coefficient from a regression of each variable against country-level fixed effects.

^b^These variables are all sourced from DHS STATcompiler (USAID and ICF-International 2017), which disaggregates variables at subnational units that we standardize across multiple DHS rounds.

### Core Results

**Fig. 1:**
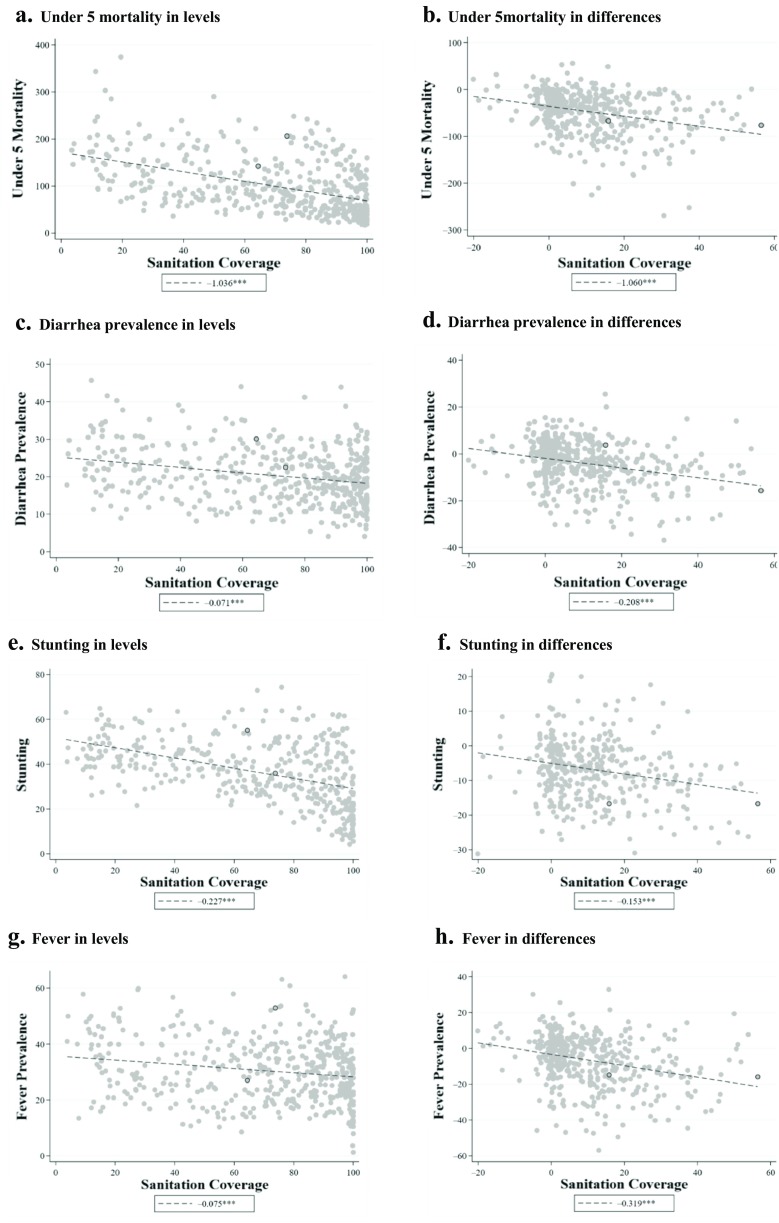
Scatter plots and slope coefficients for primary outcomes and sanitation coverage: Levels and differences. *Differences* refer to the change in an indicator from the first survey round available to the last round available; *levels* plots use data from the median survey year available for each region. Slope coefficients from linear regressions are reported in the legend of each panel. *Source:* DHS STATcompiler (USAID and ICF-International 2017). ^†^*p* < .10; *p < .05; **p < .01

**Fig. 2:**
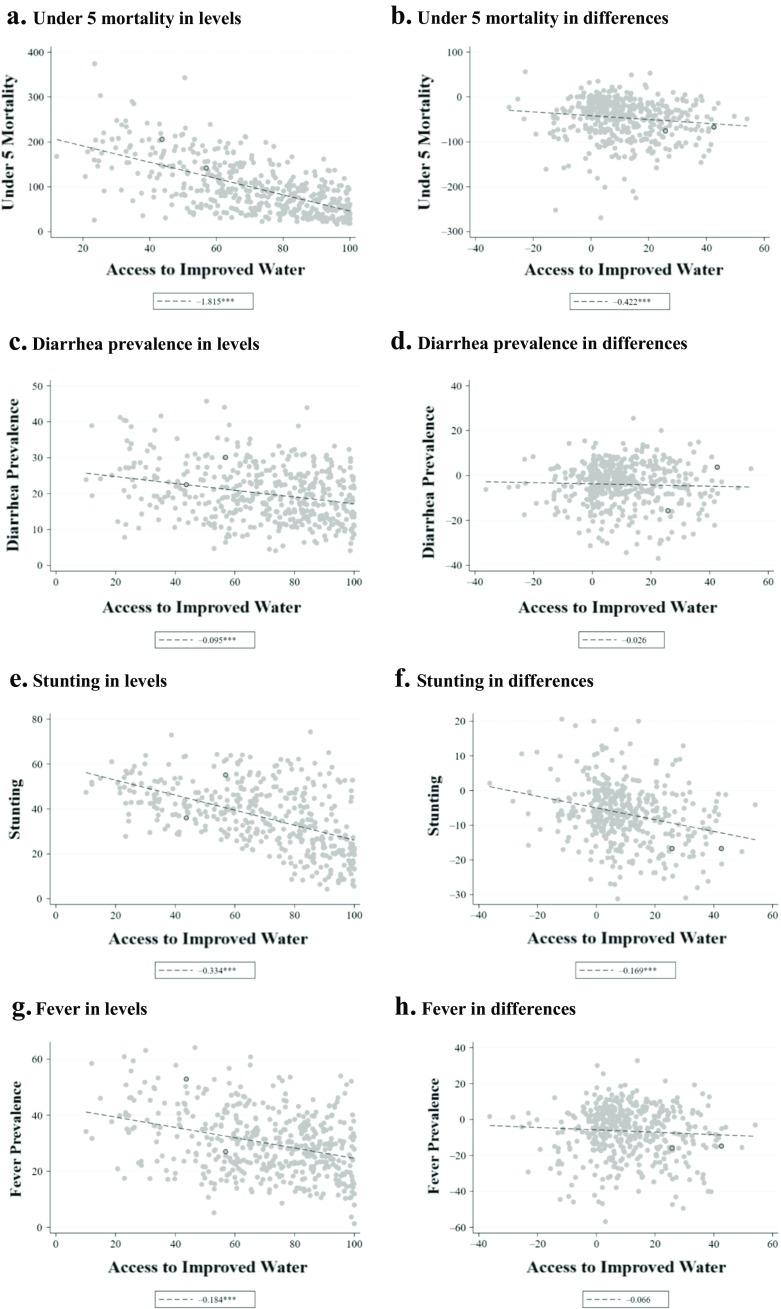
Scatter plots and slope coefficients for primary outcomes and improved water coverage: Levels and differences. *Differences* refer to the change in an indicator from the first survey round available to the last round available; *levels* plots use data from the median survey year available for each region. Slope coefficients from linear regressions are reported in the legend of each panel. *Source:* DHS STATcompiler (USAID and ICF-International 2017). ^†^*p* < .10; *p < .05; **p < .01

Have these changes in sanitation and water translated into improved child health outcomes? Figures 1 and 2 plot the mortality, diarrhea, stunting, and fever outcomes against sanitation coverage and access to improved water, respectively. For each health outcome, the left panel reports relationships in levels (cross-sectional variation), and the right panel reports these relationships for differences between the earliest completed DHS and the most recent completed DHS in each region (temporal variation). The figures therefore give some insight into the importance of netting out subnational fixed effects through differencing. For sanitation coverage, the relationships are generally negative and relatively steep in both levels and differences. However, the relationships between improved water and health outcomes are markedly weaker in differences, suggesting that the levels relationships may be confounded by fixed subnational region-level characteristics.

To investigate these associations more thoroughly, we turn to more rigorous DID models with and without adjustments for time-varying confounders and survey fixed effects. Table 2 displays the results of these regressions for under-5 mortality, diarrhea, and stunting; Table 3 does the same for wasting and fever.

**Table 2:** DID regressions of child health outcomes against sanitation and improved water

	Under-5 Mortality			Diarrhea			Stunting		
	(1)	(2)	(3)	(4)	(5)	(6)	(7)	(8)	(9)
A. Sanitation									
Households with any sanitation	–0.804**	–0.381*	–0.343*	–0.163**	–0.121**	–0.056^†^	–0.060*	–0.015	0.014
	[.000]	[.011]	[.026]	[.000]	[.000]	[.054]	[.049]	[.674]	[.673]
*R*^2^ (within)	.602	.686	.815	.196	.254	.481	.435	.516	.679
*N*	1,401	1,401	1,401	1,451	1,451	1,451	1,193	1,193	1,193
Region fixed effects	✓	✓	✓	✓	✓	✓	✓	✓	✓
Time controls	✓	✓	✓	✓	✓	✓	✓	✓	✓
Full controls		✓	✓		✓	✓		✓	✓
Survey fixed effects			✓			✓			✓
B. Water									
Households with improved water	–0.269*	–0.048	–0.118	–0.021	0.016	0.028	–0.092**	–0.042^†^	–0.037
	[.013]	[.642]	[.326]	[.335]	[.467]	[.263]	[.000]	[.064]	[.109]
*R*^2^ (within)	.589	.683	.807	.144	.237	.494	.464	.538	.703
*N*	1,479	1,479	1,479	1,515	1,515	1,515	1,176	1,176	1,176
Region fixed effects	✓	✓	✓	✓	✓	✓	✓	✓	✓
Time controls	✓	✓	✓	✓	✓	✓	✓	✓	✓
Full controls		✓	✓		✓	✓		✓	✓
Survey fixed effects			✓			✓			✓

*Note:* Numbers in brackets are *p* values.

*Source:* DHS STATcompiler (USAID and ICF-International 2017).

^†^*p* < .10; *p < .05; **p < .01

**Table 3:** WASH technology, wasting, and fever

	Wasting			Fever		
	(1)	(2)	(3)	(4)	(5)	(6)
A. Sanitation						
Households with any sanitation	–0.017	–0.023	–0.025	–0.193**	–0.163**	–0.042
	[.421]	[.295]	[.322]	[.000]	[.000]	[.272]
*R*^2^ (within)	.199	.309	.527	.426	.511	.691
*N*	1,187	1,187	1,187	1,521	1,521	1,521
Region fixed effects	✓	✓	✓	✓	✓	✓
Time controls	✓	✓	✓	✓	✓	✓
Full controls		✓	✓		✓	✓
Survey fixed effects			✓			✓
B. Water						
Households with improved water	–0.003	0.009	0.012	–0.023	0.025	–0.007
	[.870]	[.563]	[.452]	[.473]	[.446]	[.826]
*R*^2^ (within)	.234	.330	.319	.412	.510	.693
*N*	1,170	1,170	1,170	1,574	1,574	1,574
Region fixed effects	✓	✓	✓	✓	✓	✓
Time controls	✓	✓	✓	✓	✓	✓
Full controls		✓	✓		✓	✓
Survey fixed effects						✓

*Note:* Numbers in brackets are *p* values.

*Source:* DHS STATcompiler (USAID and ICF-International 2017).

**p < .01

Consistent with Fig. 1, the multivariate results in Table 2 continue to suggest an important negative relationship between changes in sanitation coverage and changes in child mortality. In the unadjusted model, a 1 percentage point increase in sanitation coverage is associated with a decrease in the under-5 mortality rate of 0.804 deaths per 1,000 births (*p* < .001). Adding the extensive set of controls somewhat attenuates the sanitation coefficient, but a meaningfully large and statistically significant relationship remains: a 1 percentage point increase in sanitation coverage predicts a reduction in under-5 child mortality of 0.381 deaths per 1,000 births (*p* = .011). Using survey fixed effects in place of the global region trends barely changes the estimate: a 1 percentage point increase in sanitation is predicted to reduce under-5 mortality by 0.343.

We also find evidence of a statistically significant relationship between sanitation coverage and diarrhea prevalence, albeit with some sensitivity to the inclusion of survey fixed effects. Without controls, a 1 percentage point increase in sanitation coverage is associated with a decrease in diarrhea prevalence of 0.163 percentage points. Adding controls reduces this association to 0.121, but both estimates have *p* values below .001. Interestingly, adding survey fixed effects reduces the magnitude of the sanitation–diarrhea association to –0.056, although it remains statistically significant at the 10 % level (*p* = .054).

For fever (Table 3), the estimate from the unadjusted and core models suggest that a 1 percentage point increase in sanitation coverage predicts a decrease in fever prevalence of 0.193 and 0.163 percentage points (*p* < .001), respectively. However, adding survey fixed effects decreases the size of the association to –0.042 and renders it statistically insignificantly different from 0 (*p* = .272). One explanation of the sensitivity of the diarrhea and fever results to survey fixed effects is that differences in survey timings explain some of the variation in these indicators because they are more likely to be influenced by seasonality (Carneiro et al. 2010).

The estimated relationship between changes in sanitation coverage and changes in stunting is highly sensitive to the inclusion of time-varying controls. In the unadjusted model, a 1 percentage point increase in sanitation coverage is associated with a modest decrease (of 0.06) in the percentage of children under 5 who are stunted (*p* = .049). Adding controls radically reduces the slope and leaves it statistically indistinguishable from 0. Replacing the global region trends with survey fixed effects also results in an association with stunting that is not statistically significantly different from 0. Table 3 also suggests no statistically significant relationship between changes in sanitation coverage and changes wasting in any of the models. We interpret this as evidence that the unconditional relationships between changes in sanitation coverage and changes in child stunting are driven by other characteristics correlated with both child stunting and WASH technology.

Estimates of the relationship between changes in access to improved water and the outcomes are shown in the bottom panels of Tables 2 and 3. The JMP/WHO indicator of access to improved water sources appears to be a substantially less important predictor of all five health and nutrition outcomes. Only stunting is significantly associated with improved water in the adjusted model without survey fixed effects, but that relationship becomes weaker (coefficient –0.037) and statistically insignificantly different from 0 (*p* = .109) when survey fixed effects are added.

### Extensions to Disaggregated Water and Sanitation Measures

As noted earlier, the literature is far from definitive about what type of water and sanitation infrastructures are likely to improve health outcomes. For sanitation, one key debate is whether toilet upgrading is the main driver of health benefits, or whether the basic elimination of open defecation via simple sanitation technologies (such as pit latrines) is paramount. We therefore disaggregate “any sanitation” into improved and unimproved sanitation categories. For water, different sources are perceived to have different levels and sources of pathogenic contaminants. But physical access to water likely also affects the prevalence of handwashing and other hygienic practices given that acquiring water from even moderately distant sources dramatically increases the implicit cost of these behaviors, and water piped to the home could generate important savings of time and effort for households (Devoto et al. 2012; Gross et al. 2018). To reflect potential differences in both contamination levels and access gradients, we therefore disaggregate the JMP/WHO definition of “any improved water” into three categories: (1) piped water to the home, (2) other piped water, and (3) nonpiped improved water access.

Results for these more disaggregated measures are reported in Tables 4 and 5. We also report the *p* value from an *F* test of whether there is no difference between each of the associations with the disaggregated WASH measures at the base of all columns.

**Table 4:** DID regressions of child health outcomes against disaggregated sanitation indicators (panel A) and against disaggregated improved water indicators (panel B)

	Under-5 Mortality			Diarrhea			Stunting		
	(1)	(2)	(3)	(4)	(5)	(6)	(7)	(8)	(9)
A. Sanitation Indicators									
Households with improved sanitation	–0.654**	–0.318*	–0.233	–0.160**	–0.120**	–0.024	–0.069*	–0.012	0.030
	[.000]	[.036]	[.144]	[.000]	[.000]	[.438]	[.036]	[.734]	[.403]
Households with unimproved sanitation	–0.813**	–0.385**	–0.366*	–0.164**	–0.121**	–0.062*	–0.060^†^	–0.015	0.011
	[.000]	[.010]	[.018]	[.000]	[.000]	[.035]	[.052]	[.672]	[.730]
*R*^2^ (within)	.608	.687	.816	.197	.254	.484	.436	.516	.679
*N*	1,401	1,401	1,401	1,451	1,451	1,451	1,193	1,193	1,193
*p* value: Improved = unimproved	.000	.081	.021	.774	.922	.009	.439	.834	.223
Region fixed effects and time controls	✓	✓	✓	✓	✓	✓	✓	✓	✓
Full controls		✓	✓		✓	✓		✓	✓
Survey fixed effects			✓			✓			✓
B. Improved Water Indicators									
Households with water piped to home	–0.437**	–0.157	–0.199	–0.01	0.076**	0.026	–0.142**	–0.107**	–0.101**
	[.002]	[.270]	[.153]	[.688]	[.009]	[.406]	[.000]	[.003]	[.001]
Households with other piped water	–0.503**	–0.14	–0.123	–0.027	0.02	0.045	–0.077*	–0.032	–0.018
	[.001]	[.303]	[.506]	[.456]	[.583]	[.223]	[.022]	[.314]	[.597]
Households with nonpiped improved water	–0.146	–0.006	–0.089	–0.022	0.004	0.023	–0.079**	–0.031	–0.02
	[.192]	[.954]	[.484]	[.306]	[.847]	[.389]	[.001]	[.188]	[.461]
*R*^2^ (within)	.595	.684	.807	.144	.244	.494	.469	.543	.706
*N*	1,479	1,479	1,479	1,515	1,515	1,515	1,176	1,176	1,176
*p* value: Piped to home = other piped	.662	.903	.664	.637	.097	.604	.072	.058	.021
*p* value: Piped to home = nonpiped	.002	.114	.288	.581	.002	.902	.006	.011	.008
*p* value: Other piped = nonpiped	.015	.257	.837	.883	.601	.513	.945	.978	.968
Region fixed effects and time controls	✓	✓	✓	✓	✓	✓	✓	✓	✓
Full controls		✓	✓		✓	✓		✓	✓
Survey fixed effects			✓			✓			✓

*Note:* Numbers in brackets are *p* values.

*Source:* DHS STATcompiler (USAID and ICF-International 2017).

^†^*p* < .10; *p < .05; **p < .01

**Table 5:** Disaggregated WASH technology, wasting, and fever

	Wasting			Fever		
	(1)	(2)	(3)	(4)	(5)	(6)
A. Sanitation						
Households with improved sanitation	–0.006	–0.019	–0.015	–0.197**	–0.172**	–0.023
	[.808]	[.439]	[.577]	[.000]	[.000]	[.599]
Households with unimproved sanitation	–0.018	–0.024	–0.027	–0.192**	–0.162**	–0.047
	[.386]	[.288]	[.286]	[.000]	[.000]	[.222]
*R*^2^ (within)	.202	.309	.528	.426	.511	.692
*N*	1,187	1,187	1,187	1,521	1,521	1,521
*p* value: Improved = unimproved	.170	.592	.224	.743	.442	.344
Region fixed effects	✓	✓	✓	✓	✓	✓
Time controls	✓	✓	✓	✓	✓	✓
Full controls		✓	✓		✓	✓
Survey fixed effects			✓			✓
B. Water						
Households with water piped to home	–0.016	–0.017	–0.039^†^	–0.026	0.054	–0.015
	[.390]	[.416]	[.090]	[.553]	[.227]	[.710]
Households with other piped water	–0.018	–0.003	–0.007	–0.091^†^	–0.009	–0.021
	[.434]	[.882]	[.792]	[.085]	[.867]	[.662]
Households with nonpiped improved water	0.007	0.018	–0.008	–0.001	0.028	0.001
	[.644]	[.254]	[.665]	[.965]	[.413]	[.981]
*R*^2^ (within)	.237	.334	.532	.414	.511	.694
*N*	1,170	1,170	1,170	1,574	1,574	1,574
*p* value: Piped to home = other piped	.931	.561	.233	.212	.211	.900
*p* value: Piped to home = nonpiped	.100	.030	.109	.508	.446	.637
*p* value: Other piped = nonpiped	.188	.281	.972	.059	.426	.637
Region fixed effects	✓	✓	✓	✓	✓	✓
Time controls	✓	✓	✓	✓	✓	✓
Full controls		✓	✓		✓	✓
Survey fixed effects			✓			✓

*Note:* Numbers in brackets are *p* values.

*Source:* DHS STATcompiler (USAID and ICF-International 2017).

^†^*p* < .10; **p < .01

The results suggest that unimproved sanitation is more robustly associated with under-5 child mortality and the prevalence of diarrhea. For both dependent variables, we reject the null of no difference between the associations at the 5 % level in specifications with survey fixed effects. Although both improved and unimproved sanitation are strongly associated with reductions in under-5 mortality in the models without survey fixed effects, the estimate for improved sanitation is no longer statistically significant at the 10 % level when survey fixed effects are included (*p* = .144). Similarly, the sanitation–diarrhea association with survey fixed effects is statistically significant for only unimproved sanitation, suggesting that a 1 percentage point increase in unimproved sanitation coverage predicts a 0.062 percentage point decrease in the number of children with diarrhea. We interpret this as evidence that eliminating open defecation via basic (“unimproved”) toilet technologies yields a larger health benefit than toilet upgrading. Neither improved nor unimproved sanitation is statistically significantly associated with stunting or wasting in any of the adjusted models, and the associations with fever are eliminated for both sanitation types when survey fixed effects are included.

The bottom panels in Tables 4 and 5 conduct the same exercise for the disaggregated water access measures. Some notable differences emerge between water piped to the home and the other two improved water types with respect to the stunting outcome. Across all three stunting specifications, water piped to the home is associated with a statistically significant reduction in stunting: a 1 percentage point increase in water piped to the home predicts roughly a 0.1 percentage point decrease in child stunting in both of the adjusted models. We find no relationship between either of the other two types of improved water sources and stunting. Changes in access to any type of improved drinking water have little association with changes in mortality or fever in the specifications with controls (Table 5). The relationships between water piped to the home and wasting or diarrhea are sensitive to the specification used: the wasting association is statistically significant only with survey fixed effects, and the diarrhea association is statistically significant—and positive—only without survey fixed effects. Given the sensitivity of these estimates to the inclusion of survey fixed effects, we are reluctant to draw any strong conclusions.

### Measurement in the Subnational Panel

The use of subnational region-level DHS data introduces distinct measurement-related advantages and potential issues relative to research using the unit-level microdata. We conduct four checks to gauge whether the use of STATcompiler subnational panel data induces problems related to the aggregated measurement of indicators.

In general, we might expect the aggregation of unit-level data to reduce the impact of classical measurement error in both the dependent and independent DHS variables by averaging out idiosyncratic unit-level measurement error. A more significant concern is that the 10-year recall period used to generate subnational mortality rate estimates could result in misclassification errors given that WASH status is reported at the time of the survey rather than at the time of death. Moreover, age disaggregation of the outcomes could be important because children’s immunity to various pathogens is typically lower in infancy and early childhood (Carneiro et al. 2010; Fisher Walker et al. 2012), and the cumulative nature of linear growth (stunting) suggests that WASH–stunting associations could be sensitive to whether children are measured in a period of rapid linear growth (i.e., *in utero* and the first two years after birth).^9^ We therefore explore whether the mortality recall period and the aggregation of outcomes for young and older children substantively affect the results.

For the mortality results, we first use STATcompiler data to disaggregate under-5 mortality into perinatal, neonatal, postneonatal, infant, and mortality between ages 1 and 5. The results in Fig. S1 of the online appendix indicate that there is no statistically significant relationship between sanitation coverage and perinatal or neonatal mortality and that approximately one-half of the overall predicted reduction in under-5 mortality comes from reductions in postneonatal mortality (ages 1–11 months), with the other one-half generated by reductions in the mortality rate among children 1–5 years of age.^10^ Online appendix Fig. S2 does the same for improved water coverage and shows that none of the associations are statistically significantly distinguishable from 0.

We next explore whether the 10-year mortality recall in the STATcompiler data is problematic through two checks. First, we restrict the analysis sample to a long panel: we retain just the first and last DHS waves conducted in each subnational region and require that these two waves be at least 10 years apart. This ensures that even the 10-year mortality rate estimates will use only those changes in the mortality outcome that occurred during the same period as the changes in WASH coverage, although the loss of two-thirds of the sample inevitably induces imprecision. Online appendix Table S5 compares the full-panel and long-panel results for postneonatal, infant, child (1–5 years), and under-5 mortality; *p* values from tests of the null hypothesis reveal that there are no differences between the estimates (shown at the base of each column). Despite the drastic difference in sample, we can never reject that the full-sample and long-panel estimates are the same, and the point estimates are qualitatively similar. Associations between the mortality rates and improved water are always close to 0 in magnitude and are never statistically significantly different from 0 for either sample. The similarity in the estimates across the two samples therefore provides some evidence that the 10-year recall is not materially affecting the results.

Second, we use DHS microdata and the synthetic cohort life table approach employed by DHS (Rutstein and Rojas 2006) to recalculate subnational region mortality rates based on 5-year and 1-year recall rather than 10-year recall. This adjustment also adds noise (which is why DHS uses 10-year recall), but if this added measurement error is uncorrelated with the regression error term, it should not induce bias. Figures S3 and S4 in the online appendix present the point estimates and 95 % confidence intervals for the sanitation and improved water access indicators, respectively.^11^ Despite the expected increase in imprecision as the recall period is reduced, the point estimates remain remarkably similar: sanitation coefficients remain negative and frequently are statistically significantly different from 0, while improved water coefficients remain close to 0 and statistically indistinguishable from 0. This finding further strengthens the argument that misalignment in the timing of the mortality rates and WASH indicators is not generating meaningful bias in the main WASH–mortality associations.

Finally, we investigate the sensitivity of the morbidity and nutrition results to age disaggregation using the DHS microdata (with appropriate survey weights) to create subnational panels for children 0–23 months and 24–59 months. This entails a sample restriction because the micro surveys and STATcompiler surveys do not perfectly align, but we confirm that our main results are robust to this restriction in the online appendix, Table S6.^12^ Table S7 (online appendix) reports separate results for children 0–23 months and 24–59 months. We find little evidence that the point estimates are sensitive to age restrictions. The sanitation coefficients are always similar in sign, magnitude, and statistical significance. The only difference for the improved water indicator is that the association between improved water and wasting is statistically significant for the 24–59 month sample only, but the difference between the wasting associations is small and not statistically distinguishable from 0. In general, age disaggregation does not materially alter the results.

### Tests for Parameter Heterogeneity

The impacts of WASH improvements on child health could systematically differ with other characteristics. We investigate two specific forms of parameter heterogeneity identified as being important in the literature. First, there may be nonlinearities in the relationships, particularly if WASH coverage generates externalities. For example, some studies have suggested that reductions in open defecation do not yield substantial benefits until sanitation coverage has reached a sufficiently high level (Andres et al. 2017; Headey et al. 2015; Jung et al. 2017). To examine whether there are nonlinearities in the WASH associations, we categorize each of the WASH access measures into indicators for whether regions were in 1 of 9 or 10 equal-sized categories: 0 % to 10 %, 10 % to 20 %, 20 % to 30 %, 30 % to 40 %, 40 % to 50 %, 50 % to 60 %, 60 % to 70 %, 70 % to 80 %, 80 % to 90 %, or 90 % to 100 % (no region has 0 % to 10 % access to improved water access).^13^ Figures S5–S12 (online appendix) display coefficient estimates and 95 % confidence intervals for the outcomes when using the binned sanitation and improved water access indicators. The results, which should be interpreted as changes in the outcome relative to regions with 0 % to 10 % sanitation coverage or 10 % to 20 % access to improved water, support the linear-in-parameters specifications in Table 2 for both WASH technologies.

Second, it has long been argued that sanitation may have larger health impacts in more densely populated areas. Hathi et al. (2017) presented the first extensive evidence of this relationship using cross-sectional variation in open defecation and the log of subnational population density to predict changes in infant mortality and child HAZ. We estimate an analogous interaction, with the difference being that we are implicitly estimating the impacts of changes in sanitation conditional on initial population density. Table S8 (online appendix) presents these results from the adjusted models with global region time trends.^14^ Similar to Hathi et al. (2017), we find evidence that the association between child HAZ and sanitation coverage is increasing in population density, although the main coefficient on “any sanitation” is sufficiently negative such that the association between sanitation coverage and HAZ turns positive only around the 90th percentile of population density. Hence, these results suggest that sanitation improvements result in modest improvements in HAZ in the highest-density regions, about one-half of which are predominantly urban areas. The sanitation–density interactions for the other health outcomes of interest generally have an unexpected sign, suggesting lower sanitation impacts in higher-density areas. Overall, then, the results do not provide strong support to the results that Hathi et al. (2017) reported.

### Assessing Identifying Assumptions

Section D of the online appendix provides a detailed description of specifications that investigate the associations between the WASH variables and other likely determinants of the main outcomes and prior trends assessments. We use these exercises to explore the possibility that the main estimates may be driven by unobserved time-varying determinants of the outcomes or variation in the outcomes that chronologically precede the observed changes in WASH coverage. The results of these specification checks, which are presented in Tables S9–S11 (online appendix), offer suggestive evidence that these two potential sources of bias are unlikely to be driving the main results. Sanitation does not predict significant variation in any of the nine other likely determinants of the main outcomes, and we fail to reject any of the null hypotheses that future sanitation coverage predicts current values of the child health and nutrition outcomes. We find a statistically significant association between improved water access and one of the nine potential determinants of the main outcomes (the likelihood of vitamin A supplementation), and there is some evidence that decreases in under-5 mortality may be associated with future increase in access to improved water access. This latter finding suggests that the improved water–mortality association in Table 2 may be biased downward.

There is some uncertainty as to whether these checks are sufficiently strong to identify evidence of bias (see online appendix, section D, for a discussion). However, the results are broadly encouraging insofar as they reveal few signs of obvious bias, particularly for the sanitation associations.

### Estimating the Impacts of Sanitation Improvements on Child Mortality Over the Millennium Development Goal Era (1990–2015)

To help put our main empirical results in context, we combine the observed changes in sanitation between 1990 and 2015 with the coefficients for under-5 mortality from Table 2 to estimate the fraction of the observed reduction in under-5 child mortality between 1990 and 2015 that can potentially be explained by sanitation improvements. Globally, sanitation coverage was estimated to have increased from 76 % to 87 % over the 1990–2015 period (WHO and UNICEF 2015), while under-5 mortality fell from 93 to 42 per 1,000 births (UNICEF 2017). The coefficient on sanitation coverage in the core under-5 mortality regression from Table 2 suggests that this 11 percentage point increase in sanitation coverage would reduce under-5 mortality by 4.19 deaths per 1,000 births,^15^ explaining approximately 8.2 % of the total observed reduction in under-5 mortality between 1990 and 2015. Thus, sanitation investments appear to have played a critical role in global efforts to reduce child mortality.

## Discussion

WASH investments are widely viewed as an integral component of improving child health outcomes in developing countries. However, experimental evaluations of WASH interventions have not always uncovered strong evidence of impacts, particularly on child nutrition outcomes, and are also potentially subject to methodological limitations related to short timeframes, poor compliance, and limited external validity. These evaluations have not been statistically powered to assess precise mortality impacts. Instead, many researchers have resorted to observational analyses that exploit cross-sectional variation in water and sanitation access. Although such studies have generated useful suggestive evidence, cross-sectional estimates may be significantly biased by omitted time-invariant factors, offer few rigorous means of gauging that bias, and do not directly address the question of whether historical changes in WASH coverage typically lead to improvements in health outcomes.

In this study, we pursue a DID analysis to address some of the limitations in both the experimental and observational literatures. The subnational panel of DHS data used herein allows us to explore longer-term changes in WASH access in a broad swathe of countries, purge regressions of important time-invariant sources of bias, conduct a range of extensions and robustness tests, and conduct several falsification exercises.

At the same time, the data and methods used in this article are subject to limitations. Although the results pass most falsification checks, we cannot definitively rule out biases from time-varying omitted variables, which would caution against drawing overly strong causal inferences from these results. Our estimates are also somewhat imprecise and are therefore subject to uncertainty in a quantitative sense. We discuss and explore potential measurement issues with the dependent variables, but another source of imprecision is measurement error in the DHS WASH indicators. Sanitation indicators in the DHS are not ideal because toilet ownership does not always equate to toilet use or to appropriate disposal of children’s stools, although the fact that we find significant and relatively large coefficients on sanitation for two of the outcomes might suggest that attenuation bias is not an overwhelming problem for sanitation. Perhaps of greater concern is that improved water infrastructure could be a poor proxy for latent water quality in a microbial sense. For example, piped water systems that lack regular and consistent water flow may become breeding grounds for pathogenic bacteria (Klasen et al. 2012). Hence, there is likely to be important unobservable heterogeneity in the quality of piped water across countries. Still, the statistically significant association between water piped into the home and stunting—and the insignificant coefficients on improved water not piped into the home—suggests that the costs associated with collecting water outside the home may have especially harmful impacts on child welfare even with heterogeneity in water quality (Gross et al. 2018).

Another limitation is that our WASH indicators solely focus on hardware measures. Improving hygiene, however, is also likely to require significant behavioral changes that are not well recorded in the DHS and similar surveys. Formal education and adult literacy programs have been shown to be associated with both health knowledge and child health more broadly (Blunch 2013, 2017; Glewwe 1999; Kovsted et al. 2003), and it may be that this kind of soft knowledge complements the availability of improved WASH hardware.

Bearing these caveats in mind, many of our results are quite consistent with the experimental WASH literature. The importance of sanitation for reducing the prevalence of diarrhea accords closely to findings from both the experimental literature (Fewtrell et al. 2005; Freeman et al. 2017; Wolf et al. 2014) and the observational literature (Fink et al. 2011). Also consistent with much of the experimental literature is the lack of any statistically significant association between changes in sanitation and changes in child stunting and wasting (Dangour et al. 2013; Freeman et al. 2017).^16^ There are plausible biological explanations for a relatively weak relationship between sanitation and stunting. Although some cohort studies have found that diarrhea episodes may contribute to stunting (Checkley et al. 2008), others have found that significant catch-up growth occurs after diarrhea episodes, thereby limiting long-run impacts on linear growth (Richard et al. 2014). Another recent line of research has speculated that animal feces may be an important contributor to EED and stunting (Headey and Hirvonen 2016; Headey et al. 2017; Mbuya and Humphrey 2016), an exposure unlikely to be influenced by conventional WASH hardware.

Despite disappointing evidence regarding sanitation’s impacts on child nutrition, we find relatively strong associations with child morbidity and mortality. We estimate that sanitation improvements have accounted for just under 10 % of the decline in child mortality from 1990 to 2015. This is a significant contribution, although because approximately 1 billion people still practice open defecation, further investments in sanitation are still very much needed.

## Electronic supplementary material

ESM 1 (PDF 1364 kb)

## Footnotes

1 The average length between the first and last survey in a country is 14 years, and the average gap between successive surveys is 5 years.

2 DHS computes subnational mortality rates using data from the 10 years preceding each survey.

3 We thank an anonymous referee for making these suggestions.

4 Table S2 (online appendix) provides the full list of DHS in each of the three data sets.

5 To ensure that changes in samples driven by the availability of control variables included in **X** and **Z** are not driving our empirical results, we impute all missing values for the controls to 0 and include dummy variables for whether the subnational region had a missing value for a given control variable. As shown in section E of the online appendix, the results are robust to alternative methods for dealing with missing controls.

6 The potential for survey fixed effects to absorb too much variation in the associations of interest arises from situations in which some regions in a country happen to have similar de-meaned values, despite having different levels or trends in the outcomes and WASH indicators.

7 Although allowing for arbitrary within-subnational region correlation in the error terms should be conservative, the results are not sensitive to clustering standard errors at the country level.

8 We also find important variation among the subnational control variables: country fixed effects typically account for just one-third to two-thirds of total variation in these controls.

9 Alderman and Headey (2018) provided evidence that stunting associations can be very sensitive to age restrictions in the sampling of child-level data.

10 The results in Figs. S1 and S2 (online appendix) are based on specifications with the full set of DHS and country-level controls and global region time trends. The results are unaffected by the inclusion of survey fixed effects in place of the global region trends.

11 The figures display results using the adjusted models with global region time trends without survey fixed effects. The analogous figures that use survey fixed effects instead of global region time trends are extremely similar although less precise in all cases.

12 We note two modest differences: the improved water–stunting association declines from –0.042 (*p* = .064) to –0.023 (*p* value = .388), and the improved water–wasting association increases from 0.009 (*p* = .563) to 0.039 (*p* = .021). Still, the results suggest that the age-disaggregated associations in the microdata sample are likely to be good estimates of what the age-specific associations would be in the full sample.

13 The categories are defined to be inclusive of the upper bound and not inclusive of the lower bound (e.g., 10 % to 20 % includes 20 % but excludes 10 %).

14 Estimates with survey fixed effects are nearly identical to the results without survey fixed effects.

15 The predicted reduction is 3.77 if we use the estimates from the specification with survey fixed effects.

16 Pickering et al. (2015) is one notable exception.

## References

[CR1] Alderman H, Headey D (2018). The timing of growth faltering has important implications for observational analyses of the underlying determinants of nutrition outcomes. PLOS ONE.

[CR2] Andres L, Briceno B, Chase C, Echenique JA (2017). Sanitation and externalities: Evidence from early childhood health in rural India. Journal of Water, Sanitation & Hygiene for Development.

[CR3] Blunch N-H (2013). Staying alive: Adult literacy programs and child mortality in rural Ghana. World Development.

[CR4] Blunch, N.-H. (2017). Adult literacy programs in developing countries. *IZA World of Labor,* 374. 10.15185/izawol.374

[CR5] Carneiro, I., Roca-Feltrer, A., Griffin, J. T., Smith, L., Tanner, M., Schellenberg, J. A., . . . Schellenberg, D. (2010). Age-patterns of malaria vary with severity, transmission intensity and seasonality in sub-Saharan Africa: A systematic review and pooled analysis. *PLOS ONE, 5*(2), e8988. 10.1371/journal.pone.0008988 10.1371/journal.pone.0008988 PMC2813874 20126547

[CR6] Center for International Earth Science Information Network (CIESIN), Columbia University; International Food Policy Research Institute (IPFRI); the World Bank; and Centro Internacional de Agricultura Tropical (CIAT). (2008). *Global Rural-Urban Mapping Project (GRUMP): Urban/rural population grids.* Palisades, NY: NASA Socioeconomic Data and Applications Center (SEDAC). Retrieved from http://sedac.ciesin.columbia.edu/gpw/index.jsp

[CR7] Checkley, W., Buckley, G., Gilman, R. H., Assis, A. M., Guerrant, R. L., Morris, S. S., . . . Black, R. E. (2008). Multi-country analysis of the effects of diarrhoea on childhood stunting. *International Journal of Epidemiology, 37,* 816–830. 10.1093/ije/dyn099 PMC2734063 18567626

[CR8] Coffey, D., Geruso, M., & Spears, D. (2016). *Sanitation, disease externalities, and anemia: Evidence from Nepal* (NBER Working Paper No. 22940). Cambridge, MA: National Bureau of Economic Research. 10.1111/ecoj.12491 PMC6001781 29937551

[CR9] Coffey D, Spears D, Vyas S (2017). Switching to sanitation: Understanding latrine adoption in a representative panel of rural Indian households. Social Science & Medicine.

[CR10] Cutler D, Miller G (2005). The role of public health improvements in health advances: The twentieth-century United States. Demography.

[CR11] Dangour, A. D., Watson, L., Cumming, O., Boisson, S., Che, Y., Velleman, Y., . . . Uauy, R. (2013). Interventions to improve water quality and supply, sanitation and hygiene practices, and their effects on the nutritional status of children. *Cochrane Database of Systematic Reviews, 8*(10). 10.1002/14651858.CD009382 10.1002/14651858.CD009382.pub2 PMC11608819 23904195

[CR12] Davis J (2004). Corruption in public service delivery: Experience from south Asia’s water and sanitation sector. World Development.

[CR13] Devoto F, Duflo E, Dupas P, Pariente W, Pons V (2012). Happiness on tap: Piped water adoption in urban Morocco. American Economic Journal: Economic Policy.

[CR14] Fewtrell, L., Kaufmann, R. B., Kay, D., Enanoria, W., Haller, L., & Colford, J. M., Jr. (2005). Water, sanitation, and hygiene interventions to reduce diarrhoea in less developed countries: A systematic review and meta-analysis. *Lancet Infectious Diseases, 5,* 42–52. 10.1016/S1473-3099(04)01253-8 15620560

[CR15] Fink G, Günther I, Hill K (2011). The effect of water and sanitation on child health: Evidence from the demographic and health surveys 1986–2007. International Journal of Epidemiology.

[CR16] Fisher Walker, C. L., Perin, J., Aryee, M. J., Boschi-Pinto, C., & Black, R. E. (2012). Diarrhea incidence in low- and middle-income countries in 1990 and 2010: A systematic review. *BMC Public Health, 12*(220). 10.1186/1471-2458-12-220 10.1186/1471-2458-12-220 PMC3323412 22436130

[CR17] Freeman, M. C., Garn, J. V., Sclar, G. D., Boisson, S., Medlicott, K., Alexander, K. T., . . . Clasen, T. F. (2017). The impact of sanitation on infectious disease and nutritional status: A systematic review and meta-analysis. *International Journal of Hygiene and Environmental Health, 220,* 928–949. 10.1016/j.ijheh.2017.05.007 28602619

[CR18] Geruso M, Spears D (2018). Neighborhood sanitation and infant mortality. American Economic Journal: Applied Economics.

[CR19] Glewwe P (1999). Why does mother’s schooling raise child health in developing countries? Evidence from Morocco. Journal of Human Resources.

[CR20] Gross E, Günther I, Schipper Y (2018). Women are walking and waiting for water: The time value of public water supply. Economic Development and Cultural Change.

[CR21] Gunther, I., & Fink, G. (2010). *Water, sanitation and children’s health: Evidence from 172 DHS surveys* (Policy Research Working Paper No. WPS5275). Washington, DC: World Bank.

[CR22] Hathi P, Haque S, Pant L, Coffey D, Spears D (2017). Place and child health: The interaction of population density and sanitation in developing countries. Demography.

[CR23] Headey, D. (2016). Are studies underestimating the effects of sanitation on child nutrition? *Lancet Global Health, 4,* e159. 10.1016/S2214-109X(15)00295-8 10.1016/S2214-109X(15)00295-8 26848090

[CR24] Headey D, Hirvonen K (2016). Is exposure to poultry harmful to child nutrition? An observational analysis for rural Ethiopia. PLOS ONE.

[CR25] Headey D, Hoddinott JF, Ali D, Tesfaye R, Dereje M (2015). The other Asian enigma: Explaining the rapid reduction of undernutrition in Bangladesh. World Development.

[CR26] Headey, D. D., Nguyen, P. H., Kim, S. S., Rawat, R., Ruel, M. T., & Menon, P. (2017). Is exposure to animal feces harmful to child nutrition and health outcomes? A multicountry observational analysis. *American Journal of Tropical Medicine and Hygiene, 96,* 961–969. 10.4269/ajtmh.16-0270 PMC5392649 27994099

[CR27] Huda TM, Unicomb L, Johnston RB, Halder AK, Yushuf Sharker MA, Luby SP (2012). Interim evaluation of a large scale sanitation, hygiene and water improvement programme on childhood diarrhea and respiratory disease in rural Bangladesh. Social Science & Medicine.

[CR28] Humphrey JH (2009). Child undernutrition, tropical enteropathy, toilets, and handwashing. Lancet.

[CR29] Jung TY, Lou W, Cheng Y (2017). Exposure-response relationship of neighborhood sanitation and children’s diarrhea. Tropical Medicine & International Health.

[CR30] Klasen S, Lechtenfeld T, Meier K, Rieckmann J (2012). Benefits trickling away: The health impact of extending access to piped water and sanitation in urban Yemen. Journal of Development Effectiveness.

[CR31] Kovsted J, Portner CC, Tarp F (2003). Child health and mortality: Does health knowledge matter?. Journal of African Economies.

[CR32] Mara D, Lane J, Scott B, Trouba D (2010). Sanitation and health. PLOS Medicine.

[CR33] Mbuya MNN, Humphrey JH (2016). Preventing environmental enteric dysfunction through improved water, sanitation and hygiene: An opportunity for stunting reduction in developing countries. Maternal & Child Nutrition.

[CR34] Ndikumana L, Pickbourn L (2017). The impact of foreign aid allocation on access to social services in sub-Saharan Africa: The case of water and sanitation. World Development.

[CR35] Pickering AJ, Djebbari H, Lopez C, Coulibaly M, Alzua ML (2015). Effect of a community-led sanitation intervention on child diarrhoea and child growth in rural Mali: A cluster-randomised controlled trial. Lancet Global Health.

[CR36] Richard, S. A., Black, R. E., Gilman, R. H., Guerrant, R. L., Kang, G., Lanata, C. F., . . . Childhood Malnutrition and Infection Network. (2014). Catch-up growth occurs after diarrhea in early childhood. *Journal of Nutrition, 144,* 965–971. 10.3945/jn.113.187161 PMC4018956 24699805

[CR37] Rutstein SO, Rojas G (2006). *Guide to DHS statistics: Demographic and health surveys methodology*.

[CR38] Schmidt W-P (2014). The elusive effect of water and sanitation on the global burden of disease. Tropical Medicine & International Health.

[CR39] Spears, D. (2013a). *How much international variation in child height can sanitation explain?* (Working paper). Princeton, NJ: Princeton University.

[CR40] Spears D (2013). Policy lessons from the implementation of India’s total sanitation campaign. India Policy Forum.

[CR41] Strunz EC, Addiss DG, Stocks ME, Ogden S, Utzinger J, Freeman MC (2014). Water, sanitation, hygiene, and soil-transmitted helminth infection: A systematic review and meta-analysis. PLOS Medicine.

[CR42] UNICEF, World Health Organization, World Bank, & UN-DESA Population Division. (2017). *Levels and trends in child mortality: Report 2017, estimates developed by the UN Inter-agency Group for Child Mortality Estimation*. New York, NY: United Nations Children’s Fund.

[CR43] USAID, & ICF-International. (2017). *DHS STATcompiler* [Data set]. Rockville, MD: ICF-International.

[CR44] Wolf, J., Prüss-Ustün, A., Cumming, O., Bartram, J., Bonjour, S., Cairncross, S., . . . Higgins, J. P. T. (2014). Systematic review: Assessing the impact of drinking water and sanitation on diarrhoeal disease in low- and middle-income settings: Systematic review and meta-regression. *Tropical Medicine & International Health, 19,* 928–942. 10.1111/tmi.12331 24811732

[CR45] Woods RI, Watterson PA, Woodward JH (1989). The causes of rapid infant mortality decline in England and Wales, 1861–1921: Part II. Population Studies.

[CR46] World Bank. (2007). *From burden to communal responsibility: A sanitation success story from southern region in Ethiopia* [English] (Sanitation and Hygiene Series Working Paper No. 38731). Washington, DC: World Bank.

[CR47] World Bank. (2017). *World Development Indicators*. Washington, DC: World Bank. Available from https://datacatalog.worldbank.org/dataset/world-development-indicators

[CR48] World Health Organization (WHO), & UNICEF (2014). *Progress on sanitation and drinking water: 2014 update*.

[CR49] World Health Organization (WHO), & UNICEF (2015). *Progress on sanitation and drinking water: 2015 update and MDG assessment*.

[CR50] Ziegelbauer K, Speich B, Mäusezahl D, Bos R, Keiser J, Utzinger J (2012). Effect of sanitation on soil-transmitted helminth infection: Systematic review and meta-analysis. PLOS Medicine.

