# Supplementary material for: Water, Sanitation, and Child Health: Evidence From Subnational Panel Data in 59 Countries
Source: Demography. 2019 Feb 28;56(2):729–52. doi: 10.1007/s13524-019-00760-y (PMC6449314; doi:10.1007/s13524-019-00760-y)
Supplement: Supplementary file 1 — (PDF 1364 kb) [file 13524_2019_760_MOESM1_ESM.pdf]

Online Appendix

Water, sanitation and child health:

Evidence from subnational panel data in 59 countries

Contents

A. Description of datasets and additional summary statistics 4

Table S1: Wave and Region Counts by Country for the main STATCOMPILER dataset 6

Table S2: DHS Microdata Samples for Nutrition and Mortality Analyses 8

Table S3: Additional Summary Statistics for Analysis Sample 14

Table S4: Summary Statistics for Dependent Variables in the Age-Disaggregated DHS data 16

B. Disaggregating by age and limiting the mortality recall window 17

Figure S1: Sanitation and age-disaggregated mortality using STATCOMPILER 18

Figure S2: Improved Water and age-disaggregated mortality using STATCOMPILER 19

Figure S3: Sanitation and mortality with limited recall periods 20

Figure S4: Improved water and mortality rate with limited recall periods 21

Table S5: WASH technology and mortality: Full sample and first and last wave results 22

Table S6: WASH technology and child health: STATCOMPILER and aggregated DHS results 23

Table S7: WASH technology, child health, and age disaggregation 24

C. Testing for parameter heterogeneity 25

Figure S5: Sanitation Coverage Categories and Under-5 Mortality 26

Figure S6: Sanitation Coverage Categories and Diarrhea Prevalence 26

Figure S7: Sanitation Coverage Categories and Stunting 27

Figure S8: Sanitation Coverage Categories and Fever Prevalence 27

Figure S9: Improved Water Access Categories and Under-5 Mortality 28

Figure S10: Improved Water Access Categories and Diarrhea Prevalence 28

Figure S11: Improved Water Access Categories and Stunting 29

Figure S12: Improved Water Access Categories and Fever Prevalence 29

Table S8: Sanitation, Population Density and Child Health Outcomes 30

D. Assessing Identifying Assumptions 31

Table S9: WASH Technology and Breastfeeding and Vaccination Behavior 38

Table S10: WASH Technology and Malaria and Vitamin A Related Behavior 39

Table S11: WASH Technology, Child Health Outcomes and Prior Trends.................................... 40

E. Sensitivity of results to alternative treatment of missing data.................................... 41

Figure S13: Outcome Associations with Sanitation Coverage and Treatment of Missing Data.... 43

Figure S14: Outcome Associations with Improved Water and Treatment of Missing Data.......... 44

A. Description of datasets and additional summary statistics

We use three subnational panels in this study. Our main data set comes from the online DHS STATCOMPILER data: https://www.statcompiler.com/en/. STATCOMPILER provides nationally and subnationally representative data for most of the standard indicators collected in the DHS. In some countries the subnational units change over time, but STATCOMPILER typically reports both the more recent and more disaggregated subnational units as well more aggregated older subnational units to allow for comparisons over a longer period of time in each country. In all cases we use the longer and more aggregated subnational units to increase the time dimension of our data. Details of the STATCOMPILER dataset are provided in Table S1.

In addition to our main dependent variables and explanatory WASH variables, we also use DHS data on a range of other common determinants of health and nutrition outcomes, and supplement subnational DHS data with country-level data of relevance from the World Development Indicators (World Bank 2017). DHS measures include housing characteristics (electricity, finished floors), maternal education (the percent of women with at least some secondary schooling), demographic indicators (total fertility rate, median birth interval), and health services used in the preceding three years (live births with no antenatal care, live births that took place in a health facility). At the national level we control for economic growth (log GDP per capita), cereal yields (a food security proxy measured as the log of kg per hectare), total health expenditures as a percent of GDP, foreign aid flows (the log of total official development aid per capita), urbanization (percent of total population in urban areas), the log of total population, and malaria incidence (per 1,000 population at risk). Descriptive statistics for these additional controls are reported in Supplement Table S3.

Two important limitations of the STATCOMPILER data are that the subnational data are not reported for children of different ages and the mortality estimates at the subnational level use a 10-year recall period to ensure there is sufficient information. To overcome these limitations, we also use multi-country micro level DHS data collated by the authors. These micro data cover most of the surveys used in

STATCOMPILER but not all. Supplement Table S2 lists the DHS included in the main analysis, the DHS included in the microaggregated sample for the nutrition and morbidity outcomes ("DHS-Nutrition" column), and the DHS included in the microaggregated sample for the mortality outcomes ("DHS-Mortality"). Table S5 in Supplement B further shows that our main STATCOMPILER-based results are robust to restricting the sample to the smallest micro data-based sample. Summary statistics for the main variables of interest in these microaggregated DHS datasets are reported in Table S4 below.

Table S1: Wave and Region Counts by Country for the main STATCOMPILER dataset

Country	Number of DHS Waves	Earliest Year	Most Recent Year	Number of Sub-National Regions
	(1)	(2)	(3)	(4)
Angola	2	2006	2011	4
Armenia	3	2000	2010	11
Bangladesh	7	1993	2014	6
Benin	4	1996	2011	6
Bolivia	4	1994	2008	11
Brazil	1	1996	1996	6
Burkina Faso	3	2003	2014	13
Burundi	2	2010	2012	5
Cambodia	4	2000	2014	16
Cameroon	4	1991	2011	5
Chad	3	1996	2014	2
Colombia	5	1990	2010	5
Comoros	2	1996	2012	3
Congo Democratic Republic	2	2007	2013	11
Cote d'Ivoire	4	1994	2011	2
Dominican Republic	6	1991	2013	14
Egypt	7	1992	2014	4
Eritrea	2	1995	2002	6
Ethiopia	3	2000	2011	11
Gabon	2	2000	2012	5
Ghana	5	1993	2014	8
Guatemala	2	1995	1998	7
Guinea	3	1999	2012	5
Guyana	2	2005	2009	2
Haiti	3	2000	2012	10
Honduras	2	2005	2011	20
India	3	1992	2005	26
Indonesia	6	1991	2012	27
Jordan	5	1997	2012	3
Kazakhstan	2	1995	1999	5
Kenya	5	1993	2014	8
Kyrgyz Republic	2	1997	2012	2
Lesotho	3	2004	2014	10
Liberia	4	2007	2013	6
Madagascar	4	1992	2008	6
Malawi	6	1992	2014	3
Mali	5	1995	2015	4
Moldova	1	2005	2005	4
Morocco	2	1992	2003	7

Mozambique	3	1997	2011	11
Namibia	3	2000	2013	12
Nepal	4	1996	2011	5
Nicaragua	2	1998	2001	17
Niger	4	1992	2012	6
Nigeria	5	1999	2013	6
Pakistan	3	1990	2012	3
Peru	9	1991	2012	4
Philippines	5	1993	2013	17
Rwanda	6	1992	2014	5
Senegal	8	1992	2014	4
Sierra Leone	2	2008	2013	4
Tanzania	6	1991	2011	9
Togo	2	1998	2013	5
Turkey	3	1993	2003	5
Uganda	4	1995	2011	4
Vietnam	2	1997	2002	10
Yemen	3	1991	2013	2
Zambia	5	1992	2013	9
Zimbabwe	4	1994	2010	10

Source: DHS STATcompiler (USAID and ICF-International 2017).

Table S2: DHS Microdata Samples for Nutrition and Mortality Analyses

Country	Year	DHS-Nutrition	DHS-Mortality
	(1)	(2)	(3)
Angola	2006	0	1
Angola	2011	0	1
Armenia	2000	1	1
Armenia	2005	1	1
Armenia	2010	1	1
Bangladesh	1993	0	1
Bangladesh	1996	1	1
Bangladesh	1999	1	1
Bangladesh	2004	1	1
Bangladesh	2007	1	1
Bangladesh	2011	1	1
Bangladesh	2014	1	1
Benin	1996	1	1
Benin	2001	1	1
Benin	2006	1	1
Benin	2011	1	1
Bolivia	1994	1	1
Bolivia	1998	1	1
Bolivia	2003	0	1
Bolivia	2008	1	1
Brazil	1986	0	0
Brazil	1996	1	1
Burkina Faso	2003	0	0
Burkina Faso	2010	0	0
Burkina Faso	2014	0	0
Burundi	2010	0	1
Burundi	2012	0	1
Cambodia	2000	1	1
Cambodia	2005	0	1
Cambodia	2010	0	1
Cambodia	2014	0	1
Cameroon	1991	0	1
Cameroon	1998	0	1
Cameroon	2004	1	1
Cameroon	2011	1	1
Chad	1996	0	1
Chad	2004	1	1
Chad	2014	0	1
Colombia	1986	0	0

Colombia	1990	0	1
Colombia	1995	1	1
Colombia	2000	1	1
Colombia	2005	1	1
Colombia	2010	1	1
Comoros	1996	1	1
Comoros	2012	1	1
Congo Democratic Republic	2007	1	1
Congo Democratic Republic	2013	1	1
Cote d'Ivoire	1994	1	1
Cote d'Ivoire	1998	1	1
Cote d'Ivoire	2005	0	1
Cote d'Ivoire	2011	1	1
Dominican Republic	1986	0	0
Dominican Republic	1991	0	1
Dominican Republic	1996	1	1
Dominican Republic	1999	0	1
Dominican Republic	2002	1	1
Dominican Republic	2007	1	1
Dominican Republic	2013	1	1
Egypt	1988	0	0
Egypt	1992	0	1
Egypt	1995	0	1
Egypt	2000	1	1
Egypt	2003	1	1
Egypt	2005	1	1
Egypt	2008	1	1
Egypt	2014	1	1
Eritrea	1995	0	0
Eritrea	2002	0	0
Ethiopia	2000	1	1
Ethiopia	2005	1	1
Ethiopia	2011	1	1
Gabon	2000	0	1
Gabon	2012	0	0
Ghana	1988	0	0
Ghana	1993	0	1
Ghana	1998	0	1
Ghana	2003	1	1
Ghana	2008	1	1
Ghana	2014	0	1
Guatemala	1987	0	0

Guatemala	1995	1	1
Guatemala	1998	1	1
Guinea	1999	1	1
Guinea	2005	1	1
Guinea	2012	1	1
Guyana	2005	0	1
Guyana	2009	1	1
Haiti	2000	1	1
Haiti	2005	1	1
Haiti	2012	1	1
Honduras	2005	1	1
Honduras	2011	1	1
India	1992	1	1
India	1998	1	1
India	2005	1	1
Indonesia	1991	0	1
Indonesia	1994	0	1
Indonesia	1997	0	1
Indonesia	2002	0	1
Indonesia	2007	0	1
Indonesia	2012	0	1
Jordan	1997	1	1
Jordan	2002	1	1
Jordan	2007	1	1
Jordan	2009	1	1
Jordan	2012	1	1
Kazakhstan	1995	1	1
Kazakhstan	1999	1	1
Kenya	1989	0	0
Kenya	1993	1	1
Kenya	1998	1	1
Kenya	2003	1	1
Kenya	2008	1	1
Kenya	2014	0	1
Kyrgyz Republic	1997	1	1
Kyrgyz Republic	2012	1	1
Lesotho	2004	0	1
Lesotho	2009	0	1
Lesotho	2014	0	1
Liberia	2007	0	0
Liberia	2009	0	0
Liberia	2011	0	0

Liberia	2013	0	0
Madagascar	1992	1	1
Madagascar	1997	1	1
Madagascar	2003	1	1
Madagascar	2008	1	1
Madagascar	2011	0	1
Madagascar	2013	0	0
Malawi	1992	1	1
Malawi	2000	1	1
Malawi	2004	0	1
Malawi	2010	1	1
Malawi	2012	0	1
Malawi	2014	0	1
Mali	1987	0	0
Mali	1995	1	1
Mali	2001	0	1
Mali	2006	1	1
Mali	2012	1	1
Mali	2015	0	1
Moldova	2005	1	1
Morocco	1987	0	0
Morocco	1992	1	1
Morocco	2003	1	1
Mozambique	1997	1	1
Mozambique	2003	0	1
Mozambique	2009	0	0
Mozambique	2011	1	1
Namibia	2000	1	1
Namibia	2006	1	1
Namibia	2013	1	1
Nepal	1996	1	1
Nepal	2001	1	1
Nepal	2006	1	1
Nepal	2011	0	0
Nicaragua	1998	1	1
Nicaragua	2001	1	1
Niger	1992	0	1
Niger	1998	1	1
Niger	2006	1	1
Niger	2012	1	1
Nigeria	1999	0	0
Nigeria	2003	1	1

Nigeria	2008	1	1
Nigeria	2010	0	1
Nigeria	2013	1	1
Nigeria	2015	0	1
Pakistan	1990	1	1
Pakistan	2006	0	1
Pakistan	2012	1	1
Peru	1986	0	0
Peru	1991	1	1
Peru	1996	1	1
Peru	2000	1	1
Peru	2004	0	0
Peru	2007	1	1
Peru	2009	1	1
Peru	2010	1	1
Peru	2011	1	1
Peru	2012	1	1
Philippines	1993	0	1
Philippines	1998	0	1
Philippines	2003	0	1
Philippines	2008	0	1
Philippines	2013	0	1
Rwanda	1992	1	1
Rwanda	2000	1	1
Rwanda	2007	1	1
Rwanda	2010	0	1
Rwanda	2013	0	1
Rwanda	2014	0	1
Senegal	1986	0	0
Senegal	1992	1	1
Senegal	1997	0	1
Senegal	2005	1	1
Senegal	2006	0	1
Senegal	2008	0	1
Senegal	2010	1	1
Senegal	2012	1	1
Senegal	2014	0	1
Sierra Leone	2008	1	1
Sierra Leone	2013	1	1
Tanzania	1991	0	0
Tanzania	1996	1	1
Tanzania	2003	0	0

Tanzania	2004	1	1
Tanzania	2007	0	1
Tanzania	2010	1	1
Tanzania	2011	0	1
Togo	1988	0	0
Togo	1998	1	1
Togo	2013	0	1
Turkey	1993	1	1
Turkey	1998	1	1
Turkey	2003	0	1
Uganda	1988	0	0
Uganda	1995	1	1
Uganda	2000	1	1
Uganda	2006	1	1
Uganda	2011	1	1
Vietnam	1997	0	1
Vietnam	2002	0	1
Yemen	1991	1	1
Yemen	1997	0	0
Yemen	2013	1	1
Zambia	1992	1	1
Zambia	1996	0	1
Zambia	2001	1	1
Zambia	2007	1	1
Zambia	2013	1	1
Zimbabwe	1988	0	0
Zimbabwe	1994	1	1
Zimbabwe	1999	1	1
Zimbabwe	2005	1	1
Zimbabwe	2010	1	1

Source: DHS STATcompiler (USAID and ICF-International 2017).

Table S3: Additional Summary Statistics for Analysis Sample

	Obs	Within-country variation (%)[a]	Mean	Percentiles 25th	50th	75th
Other Potential Determinants of the Main Outcomes[b]						
Breastfed within 1 hour of birth	1382	52.2	47.0	32.8	48.2	61.8
Received all 8 basic vaccinations	1523	51.6	59.0	44.9	62.3	75.7
Received no vaccinations	1514	68.3	8.6	1.5	4.5	11.0
Pregnant women: slept under bednet last night	499	44.8	39.3	16.5	36.6	61.4
Pregnant women: slept under ITN last night	499	52.5	29.2	6.0	24.8	47.1
Children under 5: slept under bednet last night	504	43.6	39.5	16.9	37.7	60.3
Children under 5: slept under ITN last night	504	52.5	29.3	5.8	25.1	49.0
Women took SP/Farsider during pregnancy	418	50.9	37.1	6.3	31.3	64.5
Children 6-59 mo: Received vitamin A in last 6 mo.	644	30.6	56.2	38.8	60.5	74.2
DHS Control Variables[b]						
Women with some secondary education	1621	30.7	38.6	17.4	34.8	56.5
Households with electricity	1575	33.5	47.2	12.4	44.8	80.0
Households with finished floors	1591	50.6	46.4	21.2	43.5	71.7
Total fertility rate 15-44	1644	32.8	4.1	2.9	3.9	5.3
Median birth interval (months)	1639	51.6	36.3	31.2	34.4	39.2
Place of delivery: Health facility	1567	46.2	53.1	28.9	52.2	78.1
No antenatal care	1587	44.6	15.1	2.9	7.1	19.4
WDI Control Variables[c]						
Ln Cereal Yield (kg/ha)	1636	N/A	7.5	7.1	7.4	7.9
Ln GDP per capita (US$)	1640	N/A	6.7	6.0	6.7	7.2
Total health expenditure (% of Total GDP)	1404	N/A	5.3	4.0	5.0	6.1
Ln Total ODA per capita (US$)	1636	N/A	3.1	2.3	3.6	4.1
Urban population (% of total)	1640	N/A	39.0	26.1	37.6	48.4
Ln Total Population	1640	N/A	17.1	16.0	16.7	18.2
Malaria cases per 1,000 at risk	267	N/A	176.9	29.8	105.9	207.0
Other DHS Characteristics[b,d]						
Child Height-for-age Z	1091	43.6	-1.4	-1.8	-1.4	-1.0
Region population density (people per sq. km)	1644	92.8	541.6	35.2	92.7	291.0
Children with any anemia	646	24.5	59.1	46.0	59.6	72.2
Mortality Rates[b]						
Perinatal (per 1,000 pregnancies)	1062	57.5	30.8	21.0	29.0	39.0
Neonatal (per 1,000 live births)	1499	52.8	30.8	20.0	29.0	39.0
Post-Neonatal (per 1,000 neonatal survivors)	1497	50.4	30.1	15.0	27.0	40.0
Infant (per 1,000 live births)	1497	48.7	61.0	39.0	57.0	77.0
Child (1-5 yrs) (per 1,000 1 year-olds)	1497	34.3	35.9	11.0	25.0	51.0

Note:
a. This indicator reports the share of total variation in the subnational panel explained by intra-country variation. It is equal to 100 minus the R-squared coefficient from a regression of each variable against country-level fixed effects.
b. These variables are all sourced from DHS STATcompiler (USAID and ICF-International 2017), which disaggregates variables at subnational units that we standardize across multiple DHS rounds.
c. These variables are all sourced from the World Bank's (2017) World Development Indicators.
d. Regional level population density data is sourced from Hathi et al. (2017) and the GRUMP (2008) database.
e. Calculated using DHS microdata and DHS survey weights.

Table S4: Summary Statistics for Dependent Variables in the Age-Disaggregated DHS data

	Obs	Within-country variation (%)[a]	Mean	Percentiles 25th	50th	75th
Microdata Age-Disaggregated Outcomes[e]						
Children with diarrhea (under 2)	938	57.6	21.7	15.3	21.1	27.8
Children with diarrhea (3 to 5)	938	71.8	11.9	7.5	10.6	15.3
Children stunted (under 2)	931	40.5	28.6	19.4	28.3	37
Children stunted (3 to 5)	931	36.6	41.3	27	41.5	55.2
Children with fever (under 2)	921	62.6	30.2	20.6	29.1	39
Children with fever (3 to 5)	921	68.1	24.2	15.9	22.8	31.4
Children wasted (under 2)	925	37.7	11.1	4.6	9.5	16.1
Children wasted (3 to 5)	925	44.9	6	1.7	4.4	8.6
Microdata Mortality Rates[e]						
Post-Neonatal mortality rate (5-year data)	1,467	60.8	26.5	12.4	22.8	36.1
Post-Neonatal mortality rate (1-year data)	1,467	71.4	28.1	10.2	23	40.2
Infant mortality rate (5-year data)	1,467	58.9	54	33.1	49.5	71.7
Infant mortality rate (1-year data)	1,467	68.1	56.2	28.7	52.9	78.1
Child mortality rate (5-year data)	1,467	40.9	31.2	8.9	21.3	42.7
Child mortality rate (1-year data)	1,467	48.5	34.7	7.7	22	50.3
Under 5 mortality rate (5-year data)	1,467	48	82.8	42.5	71.5	111.4
Under 5 mortality rate (1-year data)	1,467	53.5	88.2	41.5	76.1	123.5

Notes:
a. This indicator reports the share of total variation in the subnational panel explained by intra-country variation. It is equal to 100 minus the R-squared coefficient from a regression of each variable against country-level fixed effects.
b. These variables are all sourced from DHS STATcompiler (USAID and ICF-International 2017), which disaggregates variables at subnational units that we standardize across multiple DHS rounds.
c. These variables are all sourced from the World Bank's (2017) World Development Indicators.
d. Regional level population density data is sourced from Hathi et al. (2017) and the GRUMP (2008) database.
e. Calculated using DHS microdata and DHS survey weights.
*, ** and *** refer to significance at the 10%, 5% and 1% levels. P-values are reported in brackets. Source: DHS STATcompiler (USAID and ICF-International 2017).

B. Disaggregating by age and limiting the mortality recall window

The main text only considers the relationship between WASH access and under-5 mortality. However, the DHS data permit us to disaggregate under-5 mortality into mortality rates for more narrowly defined age groups. Specifically, we separately estimate the relationship between sanitation coverage and perinatal (deaths between 22 weeks gestation and one week post-partum), neonatal (first month after birth), post-neonatal (1-11 months), infant (0-11 months), and child mortality (12-59 months). Though we do not interpret the age-specific mortality relationships as being driven purely by differences in sanitation coverage at that age—accumulated exposure to open defecation at earlier life stages is likely to influence later mortality—the pattern we observe in the age-specific mortality associations may help to shed light on the potential mechanisms driving the overall sanitation-mortality link.

Figure S1 displays age-disaggregated sanitation-mortality relationships (with 95% confidence intervals) from estimating the core model with continent-specific time trends. We find no statistically significant association between sanitation coverage and either perinatal or neonatal mortality. Given that, on average, perinatal and neonatal mortality rates are higher than those measured later in infancy and childhood, the small point estimates and lack of a statistically significant association is particularly notable. Beginning with post-neonatal mortality, we estimate a statistically significant relationship between sanitation coverage and all the remaining mortality measures. Half of the overall predicted reduction in under-5 mortality appears to be from reductions in post-neonatal mortality (1-11 months), with the other half coming from a reduction in the mortality rate among children 1-5 years of age.

Figure S2 displays the same results for the access to improved water measure. We are never able to reject that the association between improved water and mortality is statistically significantly different from zero, reinforcing the lack of a statistically significant association between improved water and the aggregate under-5 mortality measure show in Table 2.

Figure S1: Sanitation and age-disaggregated mortality using STATCOMPILER

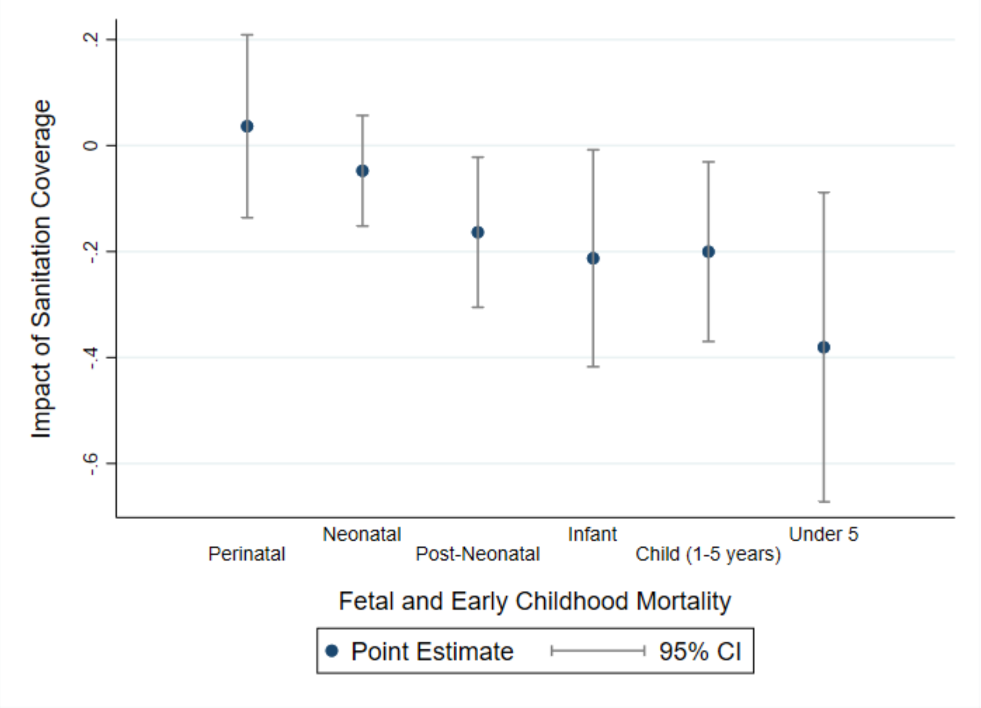

Note: Figure presents the point estimates and 95% confidence intervals for the sanitation coverage indicator and different measures of fetal and early childhood mortality. Perinatal mortality covers the period between 22 weeks gestation and 1 week post-partum, neonatal mortality includes mortality during the first month, post-neonatal mortality covers 1-11 months, infant mortality includes deaths between birth and 12 months, child (1-5 years) includes deaths between 12 and 60 months, and under five mortality includes all deaths before 60 months. Confidence intervals are based on Huber-White robust standard errors clustered at the subnational region level. Source: DHS STATcompiler (USAID and ICF-International 2017).

Figure S2: Improved Water and age-disaggregated mortality using STATCOMPILER

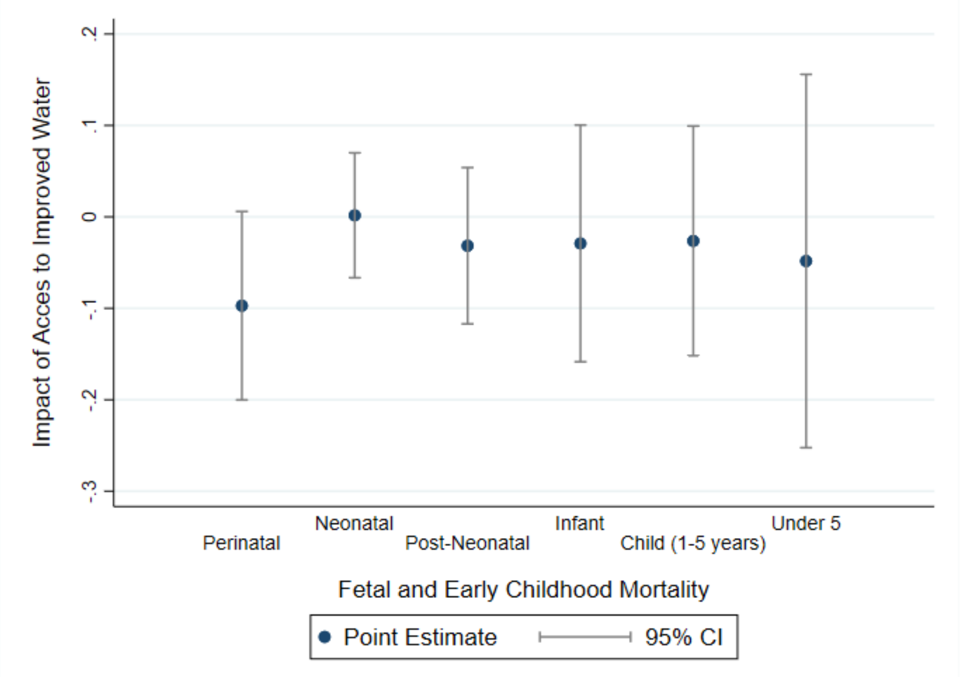

Note: Figure presents the point estimates and 95% confidence intervals for the improved water indicator and different measures of fetal and early childhood mortality. Perinatal mortality covers the period between 22 weeks gestation and 1 week post-partum, neonatal mortality includes mortality during the first month, post-neonatal mortality covers 1-11 months, infant mortality includes deaths between birth and 12 months, child (1-5 years) includes deaths between 12 and 60 months, and under five mortality includes all deaths before 60 months. Confidence intervals are based on Huber-White robust standard errors clustered at the subnational region level. Source: DHS STATcompiler (USAID and ICF-International 2017).

Figure S3: Sanitation and mortality with limited recall periods

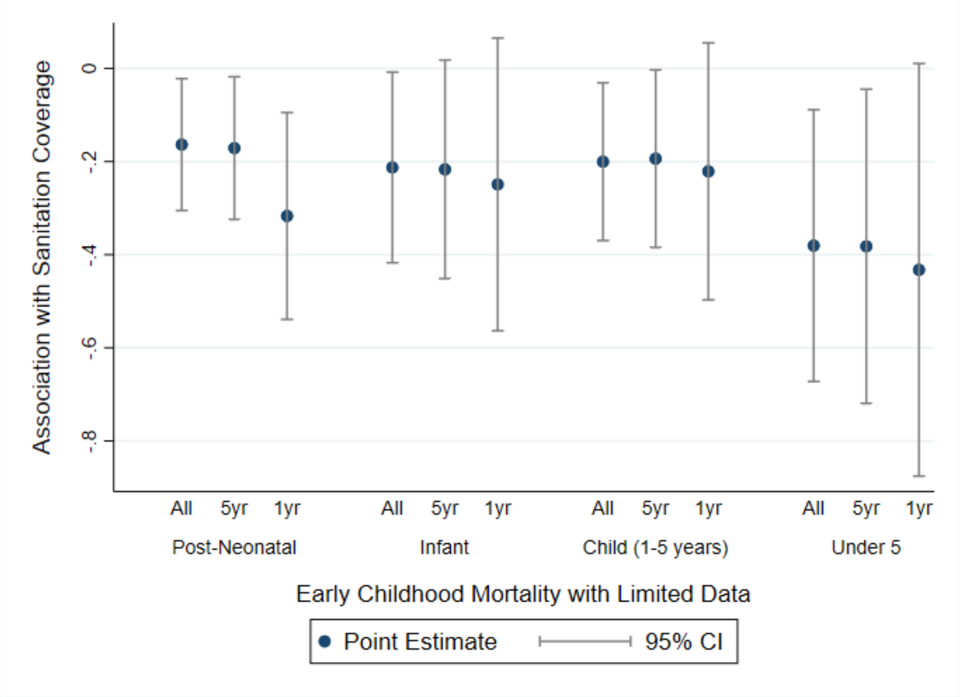

Note: Figure presents the point estimates and 95% confidence intervals for the sanitation coverage indicator with different restrictions on the years of data used to calculate the mortality rates. "All" results reproduce the adjusted models with global region time trends using ten years of data, the "5yr" results restrict the data to just the five years immediately preceding each DHS, and the "1yr" results restrict the data to just the year preceding each DHS. Dots represent point estimates and the shaded bars display the 95% confidence intervals based on standard errors clustered at the subnational region level. Source: Demographic Health Surveys (various years).

Figure S4: Improved water and mortality rate with limited recall periods

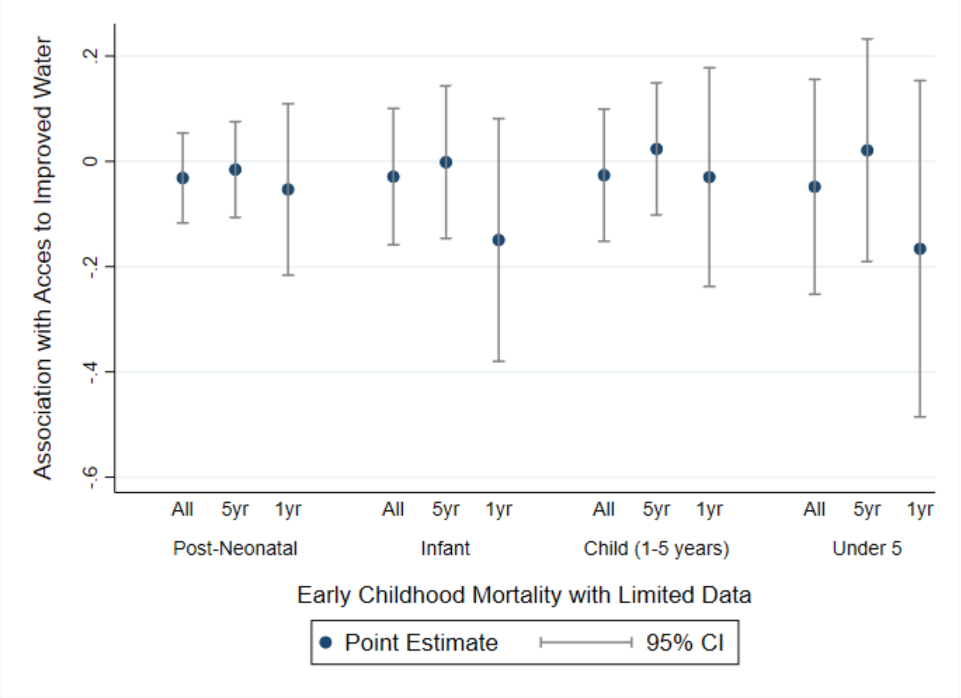

Note: Figure presents the point estimates and 95% confidence intervals for the improved water access indicator with different restrictions on the years of data used to calculate the mortality rates. "All" results reproduce the adjusted models with global region time trends using ten years of data, the "5yr" results restrict the data to just the five years immediately preceding each DHS, and the "1yr" results restrict the data to just the year preceding each DHS. Dots represent point estimates and the shaded bars display the 95% confidence intervals based on standard errors clustered at the subnational region level. Source: Demographic Health Surveys (various years).

Table S5: WASH technology and mortality: Full sample and first and last wave results

Mortality Rate	Post-Neonatal		Infant		Child (1-5 years)		Under 5	
	Full panel	First & last wave	Full panel	First & last wave	Full panel	First & last wave	Full panel	First & last wave
	(1)	(2)	(3)	(4)	(5)	(6)	(7)	(8)
Panel A								
Households with any sanitation	-0.164**	-0.149	-0.213**	-0.359	-0.200**	-0.260*	-0.381**	-0.567*
	[0.024]	[0.289]	[0.042]	[0.102]	[0.021]	[0.094]	[0.011]	[0.061]
R-squared	0.56	0.791	0.596	0.823	0.645	0.863	0.686	0.875
N	1401	537	1401	537	1401	537	1401	537
P-value: Coeff. are Equal	0.899		0.375		0.637		0.42	
Region Fixed Effects	✓	✓	✓	✓	✓	✓	✓	✓
Time Controls	✓	✓	✓	✓	✓	✓	✓	✓
Full Controls	✓	✓	✓	✓	✓	✓	✓	✓
Panel B								
Households with improved water	-0.032	0.009	-0.029	0.053	-0.026	0.026	-0.048	0.078
	[0.468]	[0.945]	[0.660]	[0.781]	[0.681]	[0.869]	[0.642]	[0.783]
R-squared	0.559	0.789	0.593	0.814	0.639	0.85	0.683	0.866
N	1479	574	1479	574	1479	574	1479	574
P-value: Coeff. are Equal	0.702		0.613		0.703		0.601	
Region Fixed Effects	✓	✓	✓	✓	✓	✓	✓	✓
Time Controls	✓	✓	✓	✓	✓	✓	✓	✓
Full Controls	✓	✓	✓	✓	✓	✓	✓	✓

Notes: "Full" columns replicate the main estimates from adjusted DID regressions with global region time trends. "Long" columns show the analogous estimates when limiting the sample to the first DHS and the last DHS for each subnational region and requiring that these DHS waves be at least ten years apart. The "P-value: Coeff are Equal" row displays p-values from tests of the null hypothesis that there is no difference between the estimates for the "Full" and "Long" samples. *, ** and *** refer to significance at the 10%, 5% and 1% levels. P-values reported in brackets. Source: DHS STATcompiler (USAID and ICF-International 2017) from various years and countries.

Table S6: WASH technology and child health: STATCOMPILER and aggregated DHS results

	Diarrhea		Stunting		Fever		Wasting	
Sample	STATCOMPILER	Micro aggregated	STATCOMPILER	Micro aggregated	STATCOMPILER	Micro aggregated	STATCOMPILER	Micro aggregated
	(1)	(2)	(3)	(4)	(5)	(6)	(7)	(8)
Panel A								
Households with any sanitation	-0.121***	-0.100***	-0.015	-0.017	-0.163***	-0.119**	-0.023	-0.019
	[0.000]	[0.004]	[0.674]	[0.663]	[0.000]	[0.020]	[0.295]	[0.531]
R-squared (within)	0.254	0.447	0.516	0.45	0.511	0.648	0.309	0.306
N	1,451	926	1,193	919	1,521	917	1187	913
Region Fixed Effects	✓	✓	✓	✓	✓	✓	✓	✓
Time Controls	✓	✓	✓	✓	✓	✓	✓	✓
Full Controls	✓	✓	✓	✓	✓	✓	✓	✓
Panel B								
Households with improved water	0.016	0.023	-0.042*	-0.023	0.025	0.062	0.009	0.039**
	[0.467]	[0.407]	[0.064]	[0.388]	[0.446]	[0.146]	[0.563]	[0.021]
R-squared (within)	0.237	0.449	0.538	0.455	0.51	0.648	0.33	0.305
N	1,515	938	1,176	931	1,574	921	1170	925
Region Fixed Effects	✓	✓	✓	✓	✓	✓	✓	✓
Time Controls	✓	✓	✓	✓	✓	✓	✓	✓
Full Controls	✓	✓	✓	✓	✓	✓	✓	✓

Notes: "Stat compiler" columns replicate the main results from Tables 2 and S5 from the adjusted DID regressions for each outcome with global region time trends. "Micro aggregated" columns show the corresponding estimates when the DHS Nutrition microdata sample is used to calculate the value for each outcome for all subnational region years in the data. *, ** and *** refer to significance at the 10%, 5% and 1% levels. P-values reported in brackets. Source: DHS and DHS STATcompiler (USAID and ICF-International 2017) from various years and countries.

Table S7: WASH technology, child health, and age disaggregation

	Diarrhea		Stunting		Fever		Wasting	
Sample:	Under 2	Age 2-5	Under 2	Age 2-5	Under 2	Age 2-5	Under 2	Age 2-5
	(1)	(2)	(3)	(4)	(5)	(6)	(7)	(8)
Panel A								
Households with any sanitation	-0.109**	-0.096***	-0.071	-0.040	-0.111**	-0.113**	-0.003	-0.03
	[0.011]	[0.005]	[0.126]	[0.323]	[0.041]	[0.036]	[0.937]	[0.331]
R-squared (within)	0.322	0.427	0.29	0.606	0.635	0.591	0.241	0.238
N	926	926	919	919	917	917	913	913
Region Fixed Effects	✓	✓	✓	✓	✓	✓	✓	✓
Time Controls	✓	✓	✓	✓	✓	✓	✓	✓
Full Controls	✓	✓	✓	✓	✓	✓	✓	✓
Panel B								
Households with improved water	0.056	0.000	-0.027	-0.003	0.061	0.059	0.030	0.041**
	[0.111]	[0.990]	[0.422]	[0.917]	[0.158]	[0.221]	[0.182]	[0.019]
R-squared (within)	0.325	0.43	0.296	0.608	0.635	0.591	0.24	0.238
N	938	938	931	931	921	921	925	925
Region Fixed Effects	✓	✓	✓	✓	✓	✓	✓	✓
Time Controls	✓	✓	✓	✓	✓	✓	✓	✓
Full Controls	✓	✓	✓	✓	✓	✓	✓	✓

Notes: "Under 2" columns show estimates from adjusted DID regressions with global region time trends using the value of the outcome calculated just using children under twenty-four months of age at the time of the survey. "Age 2-5" columns show the analogous estimates with outcomes calculated using children between the ages of twenty-four and fifty-nine months at the time of the survey. *, ** and *** refer to significance at the 10%, 5% and 1% levels. P-values reported in brackets. Source: DHS data from various years and countries.

C. Testing for parameter heterogeneity

As an extension to the main results, we categorize each of the WASH access measures into indicators for whether regions were in one of nine or ten equal sized categories—0-10%, 10-20%, 20-30%, 30-40%, 40-50%, 50-60%, 60-70%, 70-80%, 80-90%, or 90-100% —based on the percent of surveyed households in the region who reported having sanitation or access to an improved water source. There are only nine categories for access to improved water as we never observe any region with below 10% improved water access in the data. Including the full set of WASH category indicators—excluding the bottom category (0 to 10% for sanitation coverage and 10-20% for access to improved water)—relaxes the assumption that the relationship between the child health outcomes and WASH technologies is linear. This enables us to assess whether the non-linear relationships uncovered in several recent papers between sanitation and child health outcomes are relevant for our data (Headey et al. 2015; Andres et al., 2017; Jung, Lou and Cheng 2017). Supplement Figures S5-S12 display coefficient estimates and 95% confidence intervals for the outcomes when using the binned sanitation and improved water access indicators.

The results, which should be interpreted as changes in the outcome relative to regions with 0-10% sanitation coverage or 10-20% access to improved water, support the linear-in-parameters specifications in Table 2 for both WASH technologies. For all three of the outcomes for which we find a statistically significant association in Table 2 (mortality, diarrhea, fever), the figures suggest treatment effects are increasing in absolute value as sanitation coverage increases across categories. Consistent with the lack of an association for stunting and wasting, the figures for these outcomes identify flat gradients between sanitation coverage categories and the outcomes. Similarly, the association between access to improved water and child health outcomes is flat and the 95% confidence intervals never exclude zero. Therefore, for both WASH technologies, there is no evidence of non-linearities in our region-level panel.

Figure S5: Sanitation Coverage Categories and Under-5 Mortality

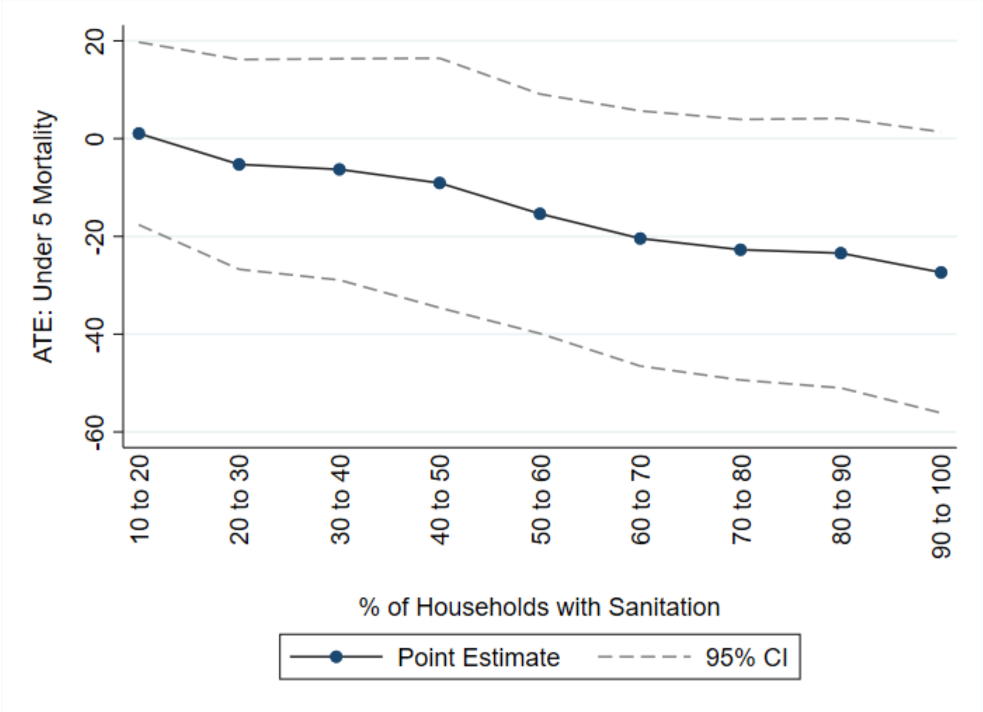

Figure S6: Sanitation Coverage Categories and Diarrhea Prevalence

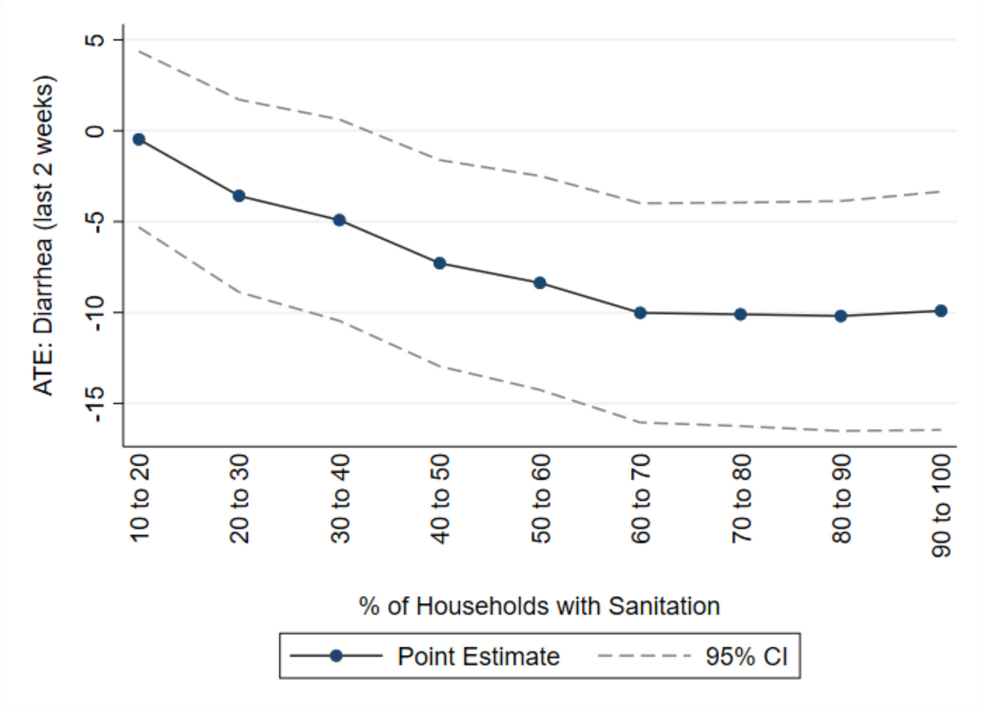

Figure S7: Sanitation Coverage Categories and Stunting

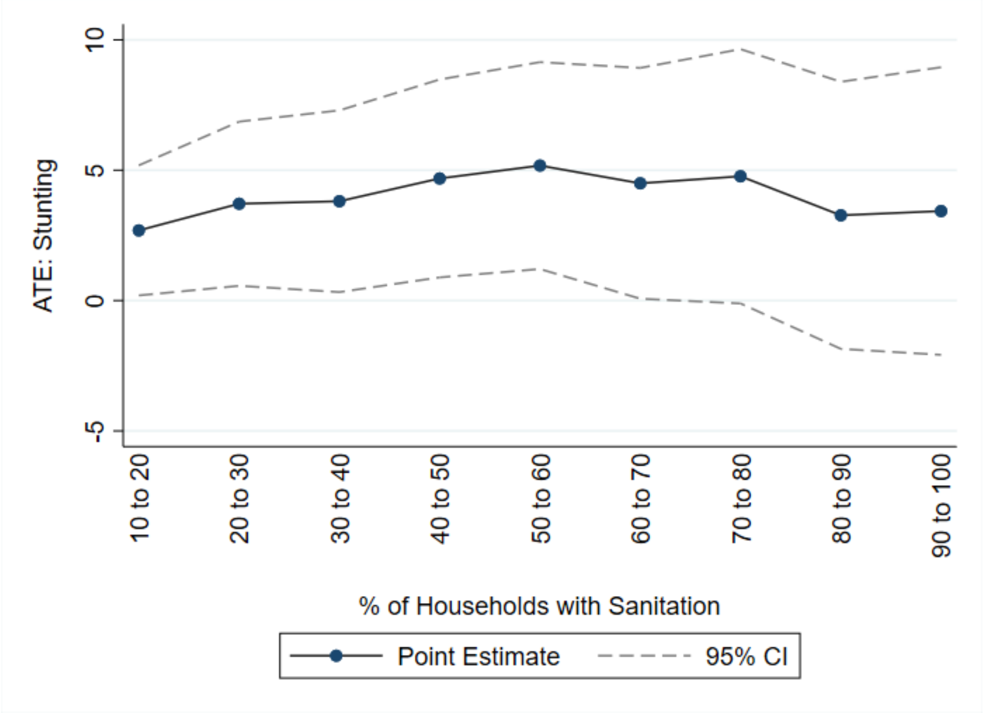

Figure S8: Sanitation Coverage Categories and Fever Prevalence

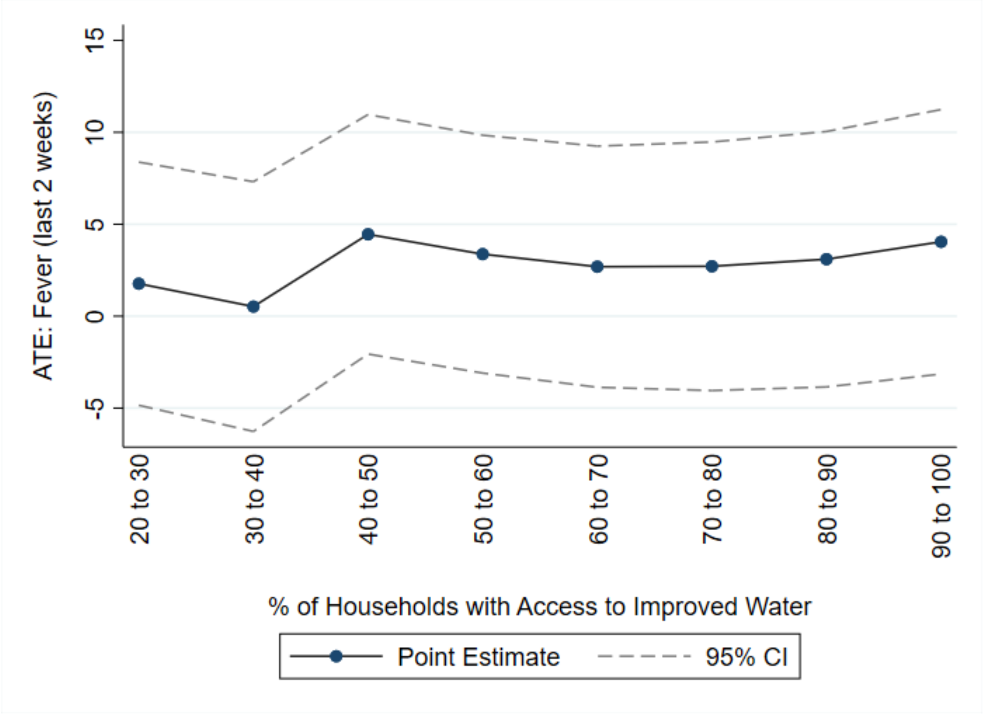

Figure S9: Improved Water Access Categories and Under-5 Mortality

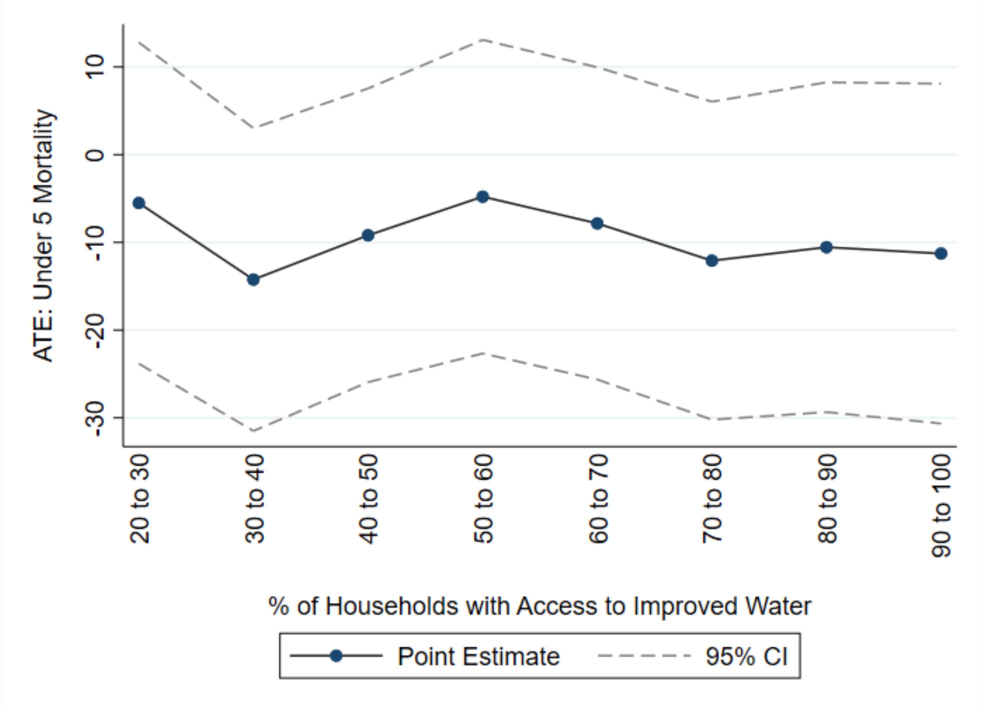

Figure S10: Improved Water Access Categories and Diarrhea Prevalence

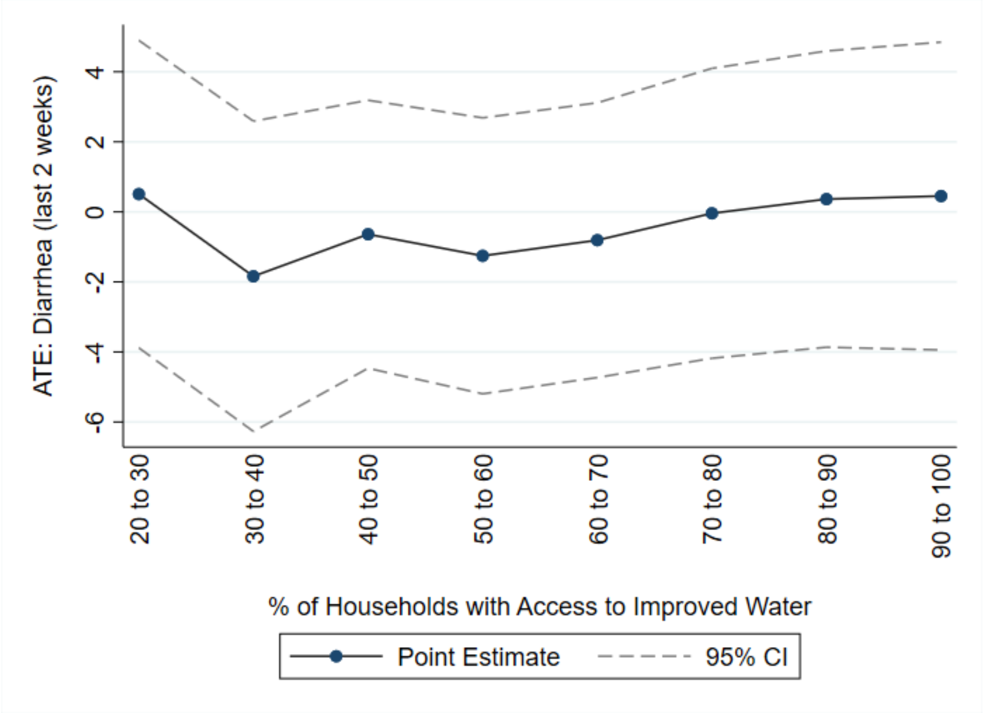

Figure S11: Improved Water Access Categories and Stunting

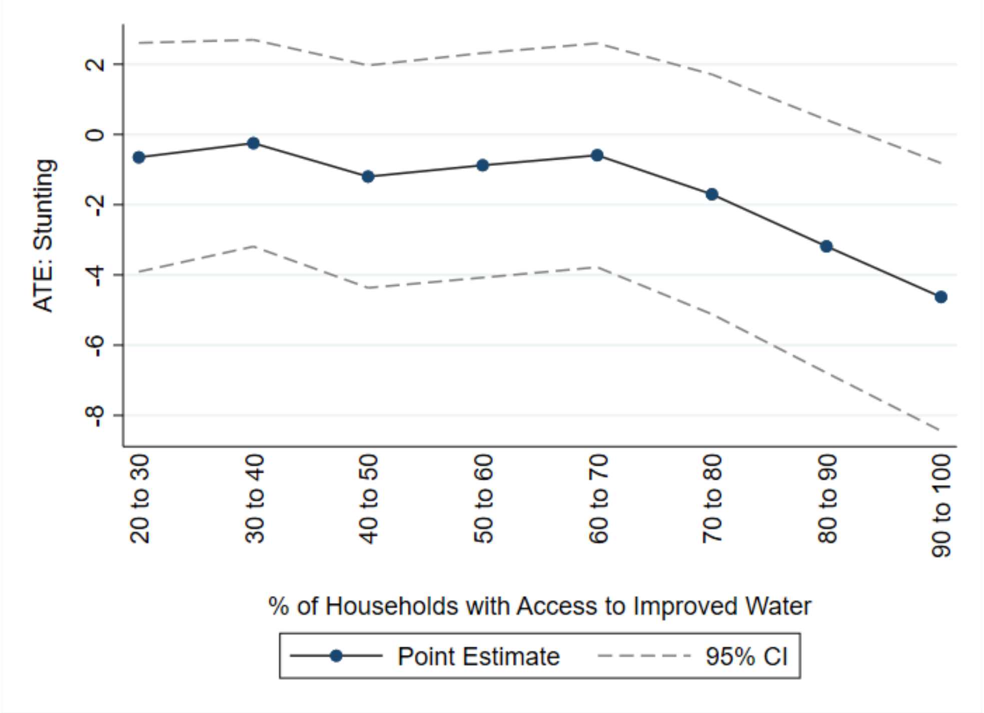

Figure S12: Improved Water Access Categories and Fever Prevalence

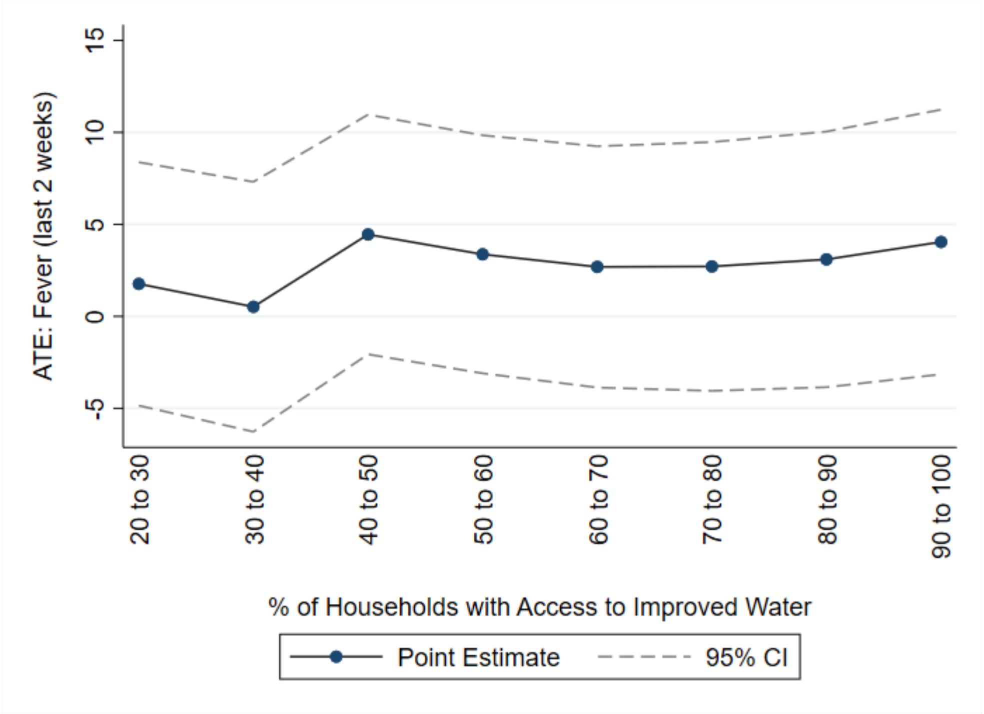

Table S8: Sanitation, Population Density and Child Health Outcomes

	Under 5 Mortality	Diarrhea	Stunting	Fever	Height-for-age	Infant Mortality
	(1)	(2)	(3)	(4)	(5)	(6)
Households with any sanitation	-0.766**	-0.210***	0.088	-0.377***	-0.008**	-0.330
	[0.018]	[0.002]	[0.161]	[0.000]	[0.015]	[0.145]
Households with any sanitation*ln Population Density	0.093	0.022	-0.024	0.053**	0.001**	0.028
	[0.139]	[0.145]	[0.101]	[0.015]	[0.035]	[0.511]
R-squared	0.687	0.256	0.519	0.515	0.539	0.597
N	1,401	1,451	1,193	1,521	1,080	1,401
Region Fixed Effects	✓	✓	✓	✓	✓	✓
Time Controls	✓	✓	✓	✓	✓	✓
Full Controls	✓	✓	✓	✓	✓	✓

Notes: *, ** and *** refer to significance at the 10%, 5% and 1% levels. P-values are reported in brackets. Source: DHS STATcompiler (USAID and ICF-International 2017).

D. Assessing Identifying Assumptions

Although the list of control variables included in the adjusted models is extensive, there is a residual concern that subnational regions exhibiting significant progress on WASH access may be making improvements in other areas relevant to child health. We assess whether the estimates from Eq. (1) are likely to be driven by omitted variable bias from unobserved time-varying factors and whether the observed changes in outcomes chronologically precede the changes in WASH access through two exercises. For the first we replace the main outcomes, $\boldsymbol{H}$, with a series of health behaviors (H^C) not included in $\boldsymbol{Z}$. Under two key assumptions discussed in more detail below, the coefficient on the WASH measures provides information about the likelihood that the main associations are driven by changes in the health behaviors or in other unobserved characteristics that are strongly correlated with the health behaviors. The second assessment examines the parallel trends assumption implicit in DID models. In our context this assumption implies that, after conditioning on controls, subnational areas that experience accelerated changes in WASH access would have had similar changes in the outcome variables in the absence of WASH accelerations. For countries with several treatment periods we can explore the plausibility of this assumption by examining whether past changes in the health outcomes predict subsequent changes in WASH coverage (Goldsmith-Pinkham, Sorkin and Swift 2017). Recovering a null estimate for these relationships boosts the credibility of the identifying assumptions by suggesting that any associations between WASH coverage and the outcomes occur in the chronologically expected order (e.g. the changes in outcomes do not precede the changes in WASH coverage).

Though neither specification check provides a broad assessment of the identifying assumptions, they offer some evidence regarding two of the more likely sources of potential bias. These exercises are described in more detail below.

Alternative Outcomes: Other Potential Determinants of the Main Outcomes

For the first specification check we replace the main dependent variables $\boldsymbol{H}$ with a series of health behaviors not included in $\boldsymbol{Z}$**.** To provide useful information about the identifying assumptions, the health behaviors we use as outcomes in these exercises (H^C) should be variables that are not directly impacted by the WASH improvements but that appropriately reflect broader improvements in healthcare and that we expect to be strongly predictive of the main outcomes of interest. For these checks we re-estimate equation (1), but after replacing the main outcomes with the health behaviors we view as likely to be predictive of the main outcomes:

$$H^C_{i,j,t} = \boldsymbol{\beta_W W}_{i,j,t} + \boldsymbol{\beta_X X}_{i,j,t} + \boldsymbol{\beta_Z Z}_{j,t} + \boldsymbol{\mu_{i,j}} + \boldsymbol{\alpha_t} + \boldsymbol{\gamma_{j,t}} + e_{i,j,t} \quad (2)$$

By assumption there should be no direct association between $\boldsymbol{W}_{i,j,t}$ and $H^C_{i,j,t}$ and there should be a strong relationship between $H^C_{i,j,t}$ and the main outcomes. Thus, rejecting that $\boldsymbol{\beta_W}$ are equal to zero suggests it is less likely that there are unobserved time-varying characteristics that are driving the results in (1) as the main outcomes, the variables included in $H^C_{i,j,t}$, and other unobserved determinants of the main outcomes should be correlated with one another.

With continuous and typically non-zero right-hand-side variables of interest, pre-treatment values of the main outcomes—which would be ideal variables to include in $H^C_{i,j,t}$—are not available. Instead, we select outcomes that we expect to be strongly related to the outcomes of interest but that should be unaffected by region-level changes in WASH coverage (Imbens and Rubin 2015): indicators of exposure to child health and nutrition interventions, such as improved initial breastfeeding practices (to capture exposure to nutritional interventions), vaccination coverage (generic child health interventions), malaria prevention and treatment indicators, and vitamin A supplementation. We note that some of these indicators are only measured for a sub-sample of observations. We do not show results for other measures of breastfeeding (e.g. median duration exclusive breastfeeding) as it seems unlikely that these would not directly be affected by changes in the WASH indicators. This is especially true for the improved water access indicator, as

households may view breastfeeding and improved water as substitutes. Supplement Table S2 shows summary statistics for all the variables in $H^C_{i,j,t}$.

While we feel the variables selected are likely to satisfy both conditions for these checks, we acknowledge that the assumption the $H^C_{i,j,t}$ outcomes are not affected by the WASH indicators after conditioning on subnational region fixed effects and the other controls is ultimately untestable. Rejecting the null hypothesis of no association between the WASH indicators and a variable in $H^C_{i,j,t}$ could therefore reflect likely bias in the main estimates or simply the possibility that changes in WASH access directly affect the alternative outcome *and* the main outcomes of interest, for example because households alter these behaviors in response to perceived changes in the risk of adverse health shocks resulting from the variation in WASH access.[1] Similarly, while there is ample evidence linking the outcomes in $H^C_{i,j,t}$ to the child health and nutrition outcomes, a failure to reject the null of no relationship between $H^C_{i,j,t}$ and one of the WASH indicators could be more likely to occur if there is attenuation bias from measurement error in the WASH variables or if an insufficient sample size critically reduces statistical power.

Despite these potential issues, our view is that these specifications provide useful information about the plausibility that there are no unobserved time-variant determinants of the main outcomes that are correlated with changes the WASH measures in the main outcome equations. If the above conditions are met—so that the variables included in $H^C_{i,j,t}$ do not respond to the changes in perceived health risk generated by variation in WASH access—but both changes in these variables and changes in WASH access are correlated with other unobserved determinants of the main outcomes (e.g. preferences for child health and nutrition outcomes), then we should expect to find positive relationships between WASH coverage and $H^C_{i,j,t}$ when we estimate equation (2).

[1] We thank an anonymous referee for pointing this out.

Supplement Tables S9 and S10 present the results of estimating (2) based on adjusted models with the global region time trends. For none of the three outcomes in Table S9 (early initiation of breastfeeding, vaccination coverage) do we estimate a statistically significant association with sanitation coverage. The relationships between sanitation coverage and the malaria and vitamin A supplementation outcomes (reported in Supplement Table S10) are similarly never statistically significantly different from zero, though the sample sizes are restricted by the limited availability of these outcomes in the DHS data. Across all 9 outcomes in $H_{i,j,t}^{C}$, we therefore never estimate a statistically significant relationship with sanitation coverage. These checks therefore suggest that there is little association between changes in sanitation coverage and changes in the other determinants of the child and nutrition outcomes included in $H_{i,j,t}^{C}$.

The analogous estimates for access to improved water are shown in the bottom panels of Tables S9 and S10. Access to improved water is statistically significantly associated with just one of the 9 measures—the likelihood that children 6-59 months of age received a vitamin A supplement during the six months preceding the survey—for which the point estimate on the improved water access measure is negative. As with the analogous checks for sanitation, we therefore estimate little evidence that changes in improved water access are statistically significantly related to other likely determinants of the main outcomes.

Parallel Trends Assessment

The second specification check we implement is a prior trends assessment that examines the parallel trends assumption implicit in DID models. In our context this assumption implies that, after conditioning on controls, subnational areas that experience accelerated changes in WASH access would have had similar changes in the outcome variables in the absence of WASH accelerations. For countries with several treatment periods we explore the plausibility of this assumption by examining whether past changes in the health outcomes predict subsequent changes in WASH coverage (Goldsmith-Pinkham, Sorkin and Swift 2017). Phrased another way, with continuous (and typically non-zero) right-hand-side variables of interest,

this amounts to exploring whether the changes in the outcomes preceded the changes in WASH coverage in the data. Recovering a null estimate for these relationships between past changes in health outcomes and future changes in WASH access boosts the credibility of the identifying assumptions by suggesting that the changes in the outcomes and the changes in WASH coverage occur in the chronologically expected order.

The conditional version of this test – as described in Goldsmith-Pinkham et al. (2017) – takes the residuals ($\widetilde{H}_{i,j,t}$) from equation (1) and estimates them as a function of sanitation coverage in the next wave:

$$\widetilde{H}_{i,j,t} = \boldsymbol{\beta_{W,t+1}} \boldsymbol{W}_{i,j,\boldsymbol{t+1}} + \boldsymbol{\mu_{i,j}} + \boldsymbol{\alpha_{t+1}} + \boldsymbol{\gamma_{j,t+1}} + u_{i,j,t} \tag{3}$$

along with controls for subnational fixed effects, a full set of survey-year dummies for the later DHS wave being used, and either DHS fixed effects or continent-specific trends for the later survey. Because the prior trends assessment focuses on exploring whether the temporal sequencing between the WASH measures and outcomes is appropriate, we use the under-5 mortality rate estimated using just the five years of data preceding each survey. The results are not sensitive to this choice.

We show results from two slightly different versions of equation (3). In the first, to ensure the sample remains constant when estimating the residuals used in (3), we code the *next* survey year ($t + 1$) to be 0 and impute the WASH measure to its year t level for the last wave in each region. The year $t + 1$ fixed effects, which therefore partial out the impact of the imputed values for the last available year, ensure that these observations do not directly impact the coefficients of interest; they are used only to estimate the residuals, through their contribution to the estimated relationship between WASH coverage and the outcomes within each year. The second method only uses data on the outcomes when the next DHS survey for that subnational region is also observed. Thus, the last DHS wave for each subnational region is not used when estimating (3). In practice, the results to not change regardless of which method is used.

While the prior trends exercise is a useful assessment of one threat to the interpretation of the main estimates, there are several caveats that are important to mention. First, the parallel trends exercise does

not provide a broad appraisal of the identifying assumptions. That is, though the failure to reject the null hypotheses of no relationships between changes in the outcomes and future changes in the WASH measures would suggest that the changes in outcomes are not likely to temporally precede the changes in WASH measures, it does not necessarily provide evidence about the likelihood that unobserved time-varying confounders are driving the associations between WASH access and the main outcomes.

Second, by relying on the subnational region DID specification to generate residuals in each period, the prior trends exercise is susceptible to unobserved time-varying sources of bias. If the predicted residuals are biased, then the subsequent associations between the predicted residuals and future WASH coverage may similarly be affected.

Third, because we do not observe future WASH for the last DHS wave, we either drop this wave from the sample used to measure the association between the current value of the outcomes and future WASH coverage or we code it to zero and we include a dummy variable for whether the value was missing. In both cases, we effectively lose one observation per subnational region. This implies that we have, on average, 3.6 DHS waves per subnational region in our data. While the main estimates are unaffected by limiting the sample to the first and last waves for each subnational region, between which the autocorrelation in the outcomes is likely to be substantially smaller (and therefore the bias due to the incidental parameters problem is also likely to be smaller), this could be more problematic when we use the shorter panel available for the prior trends assessment.

Supplement Table S11 presents the results of the conditional parallel trend test for the five child health outcomes and both WASH technology measures. Panel A displays the results for sanitation when we discard the last DHS for each region and therefore do not impute the values of future sanitation coverage and Panel B shows the results when the last DHS is included in the sample and future sanitation coverage is imputed to its value in the previous wave. Panel C and Panel D do the same for the access to improved water indicator.

Panel A uncovers no evidence that changes in the outcomes precede changes in sanitation coverage. The smallest of the five p-values is 0.310 and the point estimates are of varying signs—the estimates for under-5 mortality and wasting are positive—and small in magnitude relative to the main estimates. Panel B similarly finds no statistically significant associations between future sanitation coverage and current values of the five outcomes, with p-values ranging from 0.386 (for wasting) to 0.934 (for stunting). The point estimates are generally smaller than in Panel A and, again, are of differing signs. Both Panel A and Panel B therefore support the idea that changes in sanitation coverage are not preceded by changes in the outcomes of interest.

Panels C and D suggest there are also limited associations between future access to improved water and current diarrhea, stunting, fever, or wasting: p-values for these four outcomes range from marginally statistically insignificant (0.102 for the fever outcome when missing values for future improved water access are imputed) to nearly one (0.985 for stunting when the last DHS for each region is omitted). However, in Panel C we find a statistically significant (p-value 0.060) and negative association between future access to improved water and current under-5 mortality. While the estimate for under-5 mortality in Panel D is slightly smaller in magnitude (-0.204 as compared to -0.280) and not statistically significantly different from zero at the 10% level (p-value 0.134), both coefficients seem to suggest there may be negative trends in under-5 mortality in areas that subsequently experience increases in access to improved water. If anything, this indicates that the associations between access to improved water and under-5 mortality shown in Panel B of Table 2—which were not statistically significantly different from zero—may be more negative than the true relationship between these two variables.

Table S9: WASH Technology and Breastfeeding and Vaccination Behavior

	Early Breastfeeding (<1hr)	All 8 Vaccinations	No Vaccinations
	(1)	(2)	(3)
Panel A			
Households with any sanitation	0.111	0.095	-0.050
	[0.148]	[0.113]	[0.241]
R-squared (within)	0.419	0.507	0.529
N	1,237	1,345	1,345
Region Fixed Effects	✓	✓	✓
Time Controls	✓	✓	✓
Full Controls	✓	✓	✓
Panel B			
Households with improved water	0.085	0.032	0.054
	[0.101]	[0.465]	[0.103]
R-squared (within)	0.537	0.542	0.548
N	1,288	1,423	1,419
Region Fixed Effects	✓	✓	✓
Time Controls	✓	✓	✓
Full Controls	✓	✓	✓

Notes: *, ** and *** refer to significance at the 10%, 5% and 1% levels. P-values are reported in brackets. Source: DHS STATcompiler (USAID and ICF-International 2017).

Table S10: WASH Technology and Malaria and Vitamin A Related Behavior

	Pregnant Women		*Children Under 5*		*Women with a live birth during past 2 years*	*Children 6-59 Months*
	Slept under bednet last night	Slept under insecticide treated bednet last night	Slept under bednet last night	Slept under insecticide treated bednet last night	Took at least one dose SP/Fansidar while pregnant	Vitamin A supplement during last 6 months
	(1)	(2)	(3)	(4)	(5)	(6)
Panel A						
Households with any sanitation	0.09	0.045	0.088	-0.009	-0.09	0.146
	[0.679]	[0.821]	[0.646]	[0.961]	[0.689]	[0.285]
R-squared (within)	0.753	0.817	0.790	0.847	0.919	0.795
N	412	412	416	416	366	609
Region Fixed Effects	✓	✓	✓	✓	✓	✓
Time Controls	✓	✓	✓	✓	✓	✓
Full Controls	✓	✓	✓	✓	✓	✓
Panel B						
Households with improved water	-0.022	-0.085	0.027	0.001	0.008	-0.225***
	[0.870]	[0.531]	[0.821]	[0.993]	[0.938]	[0.006]
R-squared (within)	0.753	0.817	0.790	0.847	0.919	0.805
N	410	410	414	414	366	602
Region Fixed Effects	✓	✓	✓	✓	✓	✓
Time Controls	✓	✓	✓	✓	✓	✓
Full Controls	✓	✓	✓	✓	✓	✓

Notes: *, ** and *** refer to significance at the 10%, 5% and 1% levels. P-values are reported in brackets. Source: DHS STATcompiler (USAID and ICF-International 2017).

Table S11: WASH Technology, Child Health Outcomes and Prior Trends

	Under 5 Mortality	Diarrhea	Stunting	Fever	Wasting
	(1)	(2)	(3)	(4)	(5)
Panel A					
Future households with any sanitation	0.107	-0.04	-0.032	-0.032	0.006
	[0.475]	[0.310]	[0.475]	[0.608]	[0.867]
R-squared (within)	0.093	0.043	0.103	0.064	0.073
N	991	1042	842	1069	842
Region Fixed Effects	✓	✓	✓	✓	✓
Time Controls	✓	✓	✓	✓	✓
Full Controls	✓	✓	✓	✓	✓
Missing Future Sanitation Imputed					
Panel B					
Future households with any sanitation	0.08	-0.017	-0.003	-0.03	-0.019
	[0.537]	[0.614]	[0.934]	[0.535]	[0.386]
R-squared (within)	0.065	0.031	0.06	0.053	0.036
N	1395	1451	1193	1521	1187
Region Fixed Effects	✓	✓	✓	✓	✓
Time Controls	✓	✓	✓	✓	✓
Full Controls	✓	✓	✓	✓	✓
Missing Future Sanitation Imputed	✓	✓	✓	✓	✓
Panel C					
Future households with improved water	-0.280*	0.001	-0.034	-0.013	-0.021
	[0.060]	[0.985]	[0.219]	[0.752]	[0.413]
R-squared (within)	0.112	0.054	0.119	0.062	0.083
N	1010	1085	805	1112	805
Region Fixed Effects	✓	✓	✓	✓	✓
Time Controls	✓	✓	✓	✓	✓
Full Controls	✓	✓	✓	✓	✓
Missing Future Improved Water Imputed					
Panel D					
Future households with improved water	-0.204	-0.01	-0.004	-0.057	-0.029
	[0.137]	[0.697]	[0.862]	[0.102]	[0.120]
R-squared (within)	0.063	0.035	0.065	0.049	0.04
N	1435	1515	1176	1574	1170
Region Fixed Effects	✓	✓	✓	✓	✓
Time Controls	✓	✓	✓	✓	✓
Full Controls	✓	✓	✓	✓	✓
Missing Future Improved Water Imputed	✓	✓	✓	✓	✓

Notes: *, ** and *** refer to significance at the 10%, 5% and 1% levels. P-values are reported in brackets. Source: DHS STATcompiler (USAID and ICF-International 2017).

E. Sensitivity of results to alternative treatment of missing data

Our main results deal with the problem of missing control variables by imputing all missing values to zero and including an indicator variable for whether the value for each control was imputed for the regions in our data. We do this to preserve all available information while ensuring our sample sizes do not change because of variables other than those directly involved in one of the relationships of interest: the outcomes and WASH indicators. Though an extensive literature in statistics and the social sciences suggests this approach may lead to misleading estimates when missing data is determined by individual, household or enumerator characteristics, in our data the missing data are produced by an entirely different process. Specifically, missing data are directly determined by whether the DHS program or the World Bank elect to collect data on different indicators in a country-year. For instance, data for the malaria prevalence indicator control—the only variable in our data that is missing for more than 11% of the sample—are only available after 1999 and data for the total health expenditures as a percent of total GDP indicator—the only other control that is missing for more than 5% of the sample—is only available after 1994. Similar processes drive the missing rates for all the other controls.

While we feel our treatment of missing controls is appropriate given the reasons for missing data, we can show that our results are robust to alternative methods for dealing with the missing values. Unfortunately, multiple imputation—the most appropriate way of dealing with missing data—is not feasible in our context because when a DHS characteristic is not available, the likely correlates of that control are often also missing, and there is no variation within a country in the availability of a characteristic. Instead, we calculate estimates under two different ways of dealing with missingness: dropping the two control variables with greater than 5% missing rates—the malaria prevalence control and the total health expenditure as a percent of total GDP—and conducting complete case analysis (without including the two most frequently missing controls).

Figure S13 shows the result of this exercise for sanitation coverage while Figure S14 does the same for access to improved water. To help put the estimates under alternative methods into context, we also show the estimates from our main specifications.

For all three methods, Figure S13 displays the point estimates and 95% confidence intervals for all five outcomes. We use an M to label the main results, an NC to represent the estimates after dropping the two controls with >5% missingness rates, and a CC to represent the estimates from a complete case analysis after dropping the same two controls. For none of the five outcomes are our conclusions sensitive to the method we use to deal with missing data. Point estimates are nearly identical across the three plots for each outcome and 95% confidence intervals either do not include zero (for mortality, diarrhea, and fever) or always include zero (for stunting and wasting). Similarly, Figure S14 finds no important differences in the estimated relationship between access to improved water and the five outcomes between the three methods. Confidence intervals always include zero for the four outcomes excluding stunting and the stunting point estimate similar across the three scenarios, though the confidence interval for stunting under the complete case method does expand to include zero. Taken together, Figures S13 and S14 strongly support the idea that our method for dealing with missing controls is not importantly affecting estimates of the relationships of interest for sanitation.

Figure S13: Outcome Associations with Sanitation Coverage and Treatment of Missing Data

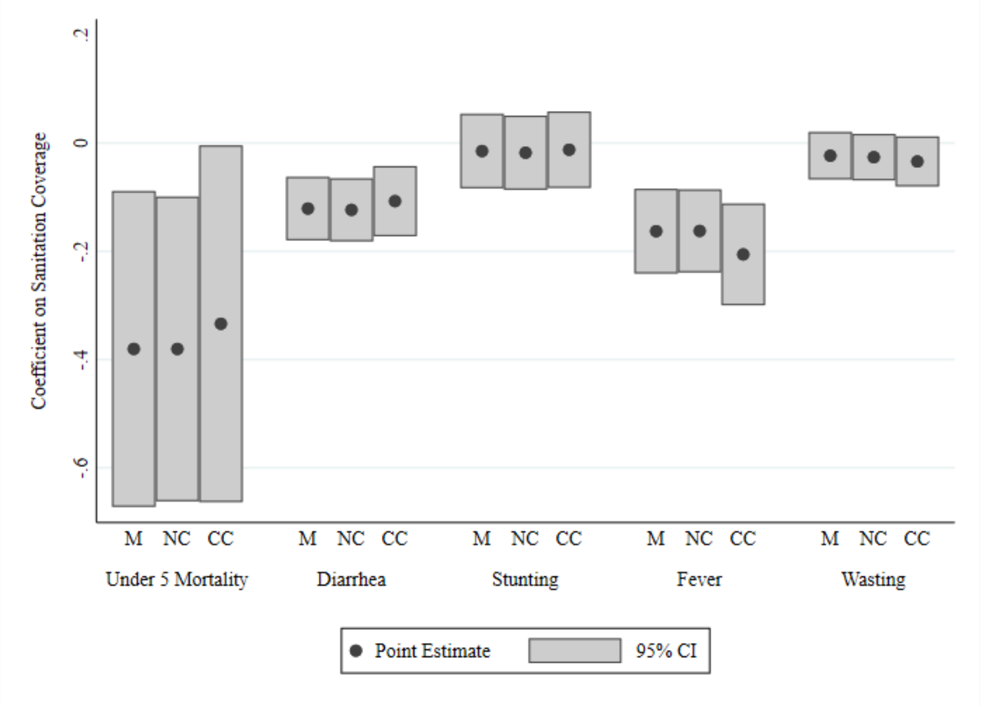

Note: Figure presents the point estimates and 95% confidence intervals for the sanitation coverage indicator under different methods of dealing for missing control variables. M represents the primary empirical results, where we impute all missing control variables to zero and include an indicator for whether each control variable was missing. NC follows the same procedure as M, but drops the WDI malaria prevalence indicator and the total expenditures as a percent of total GDP indicator (the only variables with >5% missingness) as controls. CC does complete case analysis after excluding the WDI malaria prevalence indicator and the health expenditures as a percent of total GDP as controls. Dots represent point estimates and the shaded bars display the 95% confidence intervals. Source: DHS STATcompiler (USAID and ICF-International 2017).

Figure S14: Outcome Associations with Improved Water and Treatment of Missing Data

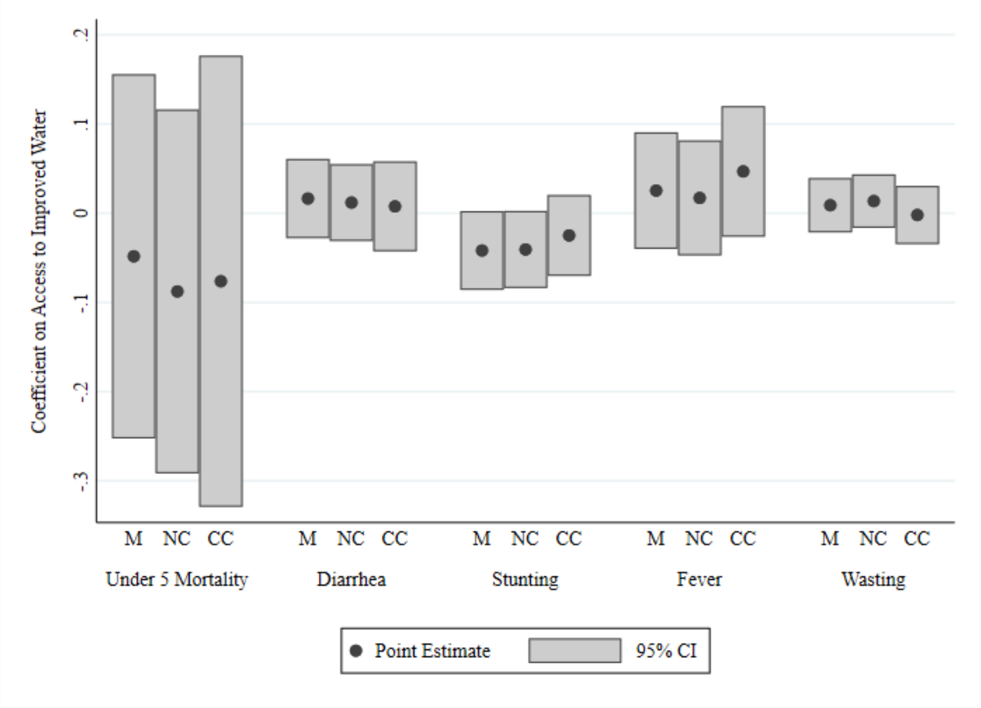

Note: Figure presents the point estimates and 95% confidence intervals for the access to improved water indicator under different methods of dealing for missing control variables. M represents the primary empirical results, where we impute all missing control variables to zero and include an indicator for whether each control variable was missing. NC follows the same procedure as M, but drops the WDI malaria prevalence indicator and the total expenditures as a percent of total GDP indicator (the only variables with >5% missingness) as controls. CC does complete case analysis after excluding the WDI malaria prevalence indicator and the health expenditures as a percent of total GDP as controls. Dots represent point estimates and the shaded bars display the 95% confidence intervals. Source: DHS STATcompiler (USAID and ICF-International 2017).
